# CRISPR/Cas9‐mediated whole genomic wide knockout screening identifies mitochondrial ribosomal proteins involving in oxygen‐glucose deprivation/reperfusion resistance

**DOI:** 10.1111/jcmm.15580

**Published:** 2020-07-02

**Authors:** Xinjie Guan, Hainan Zhang, Haiyun Qin, Chunli Chen, Zhiping Hu, Jieqiong Tan, Liuwang Zeng

**Affiliations:** ^1^ Center for Medical Genetics School of Life Sciences Central South University Changsha Hunan China; ^2^ Hunan Key Laboratory of Medical Genetics Central South University Changsha Hunan China; ^3^ Hunan Key Laboratory of Animal Model for Human Diseases Central South University Changsha Hunan China; ^4^ Department of Neurology Second Xiangya Hospital Central South University Changsha Hunan China

**Keywords:** cerebral ischaemia‐reperfusion injury, clustered regularly interspaced short palindromic repeats)/CRISPR‐associated protein 9, mitochondrial ribosomal protein, neuroprotection, oxygen‐glucose deprivation/reperfusion

## Abstract

Recanalization therapy by intravenous thrombolysis or endovascular therapy is critical for the treatment of cerebral infarction. However, the recanalization treatment will also exacerbate acute brain injury and even severely threatens human life due to the reperfusion injury. So far, the underlying mechanisms for cerebral ischaemia‐reperfusion injury are poorly understood and effective therapeutic interventions are yet to be discovered. Therefore, in the research, we subjected SK‐N‐BE(2) cells to oxygen‐glucose deprivation/reperfusion (OGDR) insult and performed a pooled genome‐wide CRISPR (clustered regularly interspaced short palindromic repeats)/Cas9 (CRISPR‐associated protein 9) knockout screen to discover new potential therapeutic targets for cerebral ischaemia‐reperfusion injury. We used Metascape to identify candidate genes which might involve in OGDR resistance. We found that the genes contributed to OGDR resistance were primarily involved in neutrophil degranulation, mitochondrial translation, and regulation of cysteine‐type endopeptidase activity involved in apoptotic process and response to oxidative stress. We then knocked down some of the identified candidate genes individually. We demonstrated that MRPL19, MRPL32, MRPL52 and MRPL51 inhibition increased cell viability and attenuated OGDR‐induced apoptosis. We also demonstrated that OGDR down‐regulated the expression of MRPL19 and MRPL51 protein. Taken together, our data suggest that genome‐scale screening with Cas9 is a reliable tool to analyse the cellular systems that respond to OGDR injury. MRPL19 and MRPL51 contribute to OGDR resistance and are supposed to be promising targets for the treatment of cerebral ischaemia‐reperfusion damage.

## 1 INTRODUCTION

Cerebral infarction is one of the prime reasons for mortality and permanent disability worldwide. Currently, the most effective treatment for cerebral infarction is recanalization of occluded vessels by intravenous thrombolysis or endovascular therapy within the time window.^1^, ^2^, ^3^ Despite re‐building, blood supply for the ischaemic region is the most urgent and important therapy for cerebral infarction, but the recanalization treatment will also lead to ischaemia‐reperfusion injury, which exacerbates acute brain injury and even severely threatens human life.

The molecular mechanisms underlying ischaemia‐reperfusion injury are multifactorial, including mitochondrial defects, oxidative stress, apoptosis, autophagy/mitophagy and neuroinflammation.^4^, ^5^, ^6^ Peroxynitrite (ONOO‐), one of the reactive nitrogen species, recruits Drp1 to damaged mitochondria and activates mitophagy excessively, which will aggravate cerebral ischaemia‐reperfusion injury.^7^, ^8^ Necroptosis and apoptosis are also be of great importance in cerebral ischaemia‐reperfusion injury.^9^ Neuroinflammation induced by microglia activation is an key factor that leads to neuron death in ischaemia‐reperfusion injury.^10^, ^11^ Mitochondrial quality control is supposed to be pivotal in cerebral ischaemia‐reperfusion damage,^12^ and dysfunction of membrane trafficking also contributes to cerebral damage in ischaemia‐reperfusion injury.^13^ Although these pathophysiological changes are critical during the development of cerebral ischaemia‐reperfusion insult, they are far from possible clinical therapeutics. Therefore, it is urgent and important to further explore the pathogenetic mechanisms underlying cerebral ischaemia‐reperfusion injury and develop effectively accessible neuroprotective strategies.

CRISPR (clustered regularly interspaced short palindromic repeats)/Cas9 (CRISPR‐associated protein 9) is an DNA endonuclease and can edit specific DNA sites that complementary to a guide RNA.^14^, ^15^ CRISPR‐Cas9 is a powerful, cutting‐edge gene editing tool. CRISPR knockout library can be personalized to contain genes that you want to knockout in single experiment. This cutting‐edge tool can help us to assess gene function and identify and validate novel drug targets or study the underlying mechanisms of human diseases.^16^ To explore the mechanisms of oxygen‐glucose deprivation/reperfusion (OGDR) resistance, genome‐scale CRISPR/Cas9 knockout (GeCKO) screening technology was employed to identify new regulators involved in OGDR resistance in human neuroblastoma cell line, SK‐N‐BE(2) cells. Moreover, mitochondrial ribosomal protein L19 (MRPL19) and MRPL51 were validated as OGDR resistance genes, indicating that they are important contributors and potential therapeutic targets for ischaemia‐reperfusion injury in ischaemic stroke.

## 2 MATERIALS AND METHODS

### 2.1 Lentiviral production of the sgRNA library

Packaging and purification of lentivirus of sgRNA library were performed as previously described.^17^ Briefly, HEK293T cells were cultured in DMEM supplemented with 10% foetal bovine serum (FBS). 4 μg of GeCKO library (#1000000048; Addgene), 2 μg of pV‐SVg (#8454; Addgene) and 6 μg of psPAX2 (#12260; Addgene) packing plasmids were cotransfected in a 10 cm dish using Lipofectamine 2000 reagent (Invitrogen) following the manufacturer's protocol. After transfection for 6 hours, the cell culture media was changed to flesh complete culture media. The media was collected after 48 hours and centrifuged at 1,000 *g* at 4°C for 20 minutes to remove the cell debris. The supernatant was filtered (0.45‐µm pore size) and concentrated by ultracentrifugation (Beckmann) at 24 000 rpm for 2 hours at 4°C. The virus preparation was finally resuspended with DMEM overnight at 4°C, divided into aliquots and stored at −80°C.

### 2.2 Lentiviral transduction of the sgRNA library

SK‐N‐BE(2) cells were cultured at 37°C, 5% CO_2_ in the DMEM containing 10% FBS (Invitrogen) with 4 mmol/L l‐glutamine, and 10 µg/mL penicillin and streptomycin. 3 × 10^8^ SK‐N‐BE(2) cells were infected with the GeCKO library viruses at a multiplicity of infection (MOI) of 0.3 to ensure that most cells receive only 1 viral construct in the presence of 10 µg/mL of polybrene. 48 hours after infection, the medium was change to fresh complete DMEM containing 1 μg/mL puromycin for 7‐day selection.

### 2.3 Oxygen‐glucose deprivation/reperfusion

To mimic ischaemic‐like conditions in vitro, SK‐N‐BE(2) cells were exposed to oxygen‐glucose deprivation (OGD) for 4 hours and then returned to 95% air, 5% CO_2_ and glucose‐containing medium for 6 hours. First, SK‐N‐BE(2) cells were transferred into a temperature‐controlled (37°C) anaerobic chamber (Forma Scientific) containing a gas mixture composed of 5% CO_2_ and 95% N_2_. The culture medium was replaced with deoxygenated glucose‐free Hanks' Balanced Salt Solution (Invitrogen), and cells were maintained in the hypoxic chamber for 4 hours. After OGD, SK‐N‐BE(2) cells were maintained in DMEM supplemented with 10% FBS under normoxic culture conditions for 6 hours.

### 2.4 OGDR resistance gene screening and DNA sequencing

SK‐N‐BE(2) cells were exposed to OGD for 4 hours and then refeed to glucose‐containing medium to recovery for 6 hours in 95% air, 5% CO_2_. The viable cells were collected. The genomic DNA was isolated from surviving cells using a Blood & Cell Culture DNA Midi Kit (Quiagen, Hilden, Germany). PCR was performed in two steps follow the protocol as described by Dr Feng Zhang. The amplicons were added by the second PCR and sequenced using a HiSeq 2500 (Illumina). The forward primer is 5′‐CTTGTGGAAAGGACGAAACA‐3′. The reverse primer is 5′‐GCCAATTCCCACTCCTTTCA‐3′. The raw sequencing data were in FASTQ form and analysed using customized GeCKO screen pipelines. Briefly, the raw sequencing reads were de‐multiplexed by using the different barcodes in the reverse primer. The processed data were removed the sequences from beginning to sgRNA priming site primers. Trimmed reads were mapped to the indexed GeCKO v2 libraries A and B. Read counts of sgRNA were quantified by Model‐based Analysis of Genome‐wide CRISPR‐Cas9 Knockout (MAGeCK) v5.6.0 for each sample. Count data of genes were filtered, normalized and ranked by MAGeCK.^18^

### 2.5 RNA interference

The oligo RNA for specific genes and non‐target was obtained from GenePharma (Shanghai, China). The oligo RNAs were transfected with Lipofectamine 2000 reagent (Invitrogen) according to protocol provide by the manufacturer. After siRNA transfection 48 hours, the cells were exposed to OGD for 4 hours and then returned to 95% air, 5% CO_2_, and glucose‐containing medium for different recovery times to induce cell apoptosis.

### 2.6 MTT assay

Cell viability was determined by 3,(4,5‐diamethylthiazol‐2‐yl)‐2,5‐diphenyltetrazolium bromide (MTT) colorimetric assay. Briefly, after siRNA transfection, 30 μL of the MTT solution was added to each well. Subsequently, cells were maintained at 37°C for 3 hours. After 3 hours, the wells were aspirated, and the plates were left to dry overnight. 50 μL of dimethyl sulphoxide (DMSO) was then added to each well and pipette up and down to dissolve the formazan MTT crystals. The plates were then shaken for 1 hour and read spectrophotometrically at 570 nm in a plate spectrophotometer. The data were recorded and were represented as per cent cell viability.

### 2.7 Apoptosis assay

After transfection for 48h, cells were washed with PBS and resuspended in binding buffer for Annexin‐V‐FITC and propidium iodide (PI) double staining with a Apoptosis Assay Kit (BD Biosciences Pharmingen, USA). Briefly, SK‐N‐BE(2) cells were collected. The collected cells were washed twice with PBS and were then added 500 μL binding buffer, 5 μL Annexin‐V‐FITC and 10 μL PI to each group. Subsequently, the cells were incubated at 37°C for 10 minutes in dark. The apoptotic cells were analysed on a flow cytometer and Cell QuestPro software (BD Biosciences).

### 2.8 Western blot

The SK‐N‐BE(2) cells were lysed by SDS sample buffer (63 mmol/L Tris HCl, 10% Glycerol, 2% SDS) with protease and phosphatase inhibitors (Sigma). 20 μg of total protein was loaded in a SDS/polyacrylamide (SDS/PAGE) gel and electrophoresed. The proteins subsequently transferred onto a PVDF membrane. The membrane was blocked in TBS‐Tween buffer (20 mmol/L TrisHCl, 5% non‐fat milk, 150 mmol/L NaCl and 0.05% Tween‐40) for 3 minutes at room temperature (RT). Thereafter, the PVDF membrane was incubated with primary rabbit MRPL19 and MRPL51 antibodies (1:1000 dilution, Novus Biologicals) at 4°C overnight. The membrane was washed with TBST for three times and then incubated with the secondary antibodies conjugated with horseradish peroxidase (HRP) for 1 hour at RT. Bands were visualized via an enhanced chemiluminescence kit (ECL) according to protocol provided by the manufacturer (GE Health).

### 2.9 Data and statistical analysis

Metascape was used to identify gene enrichment terms in genes with the number of unique sgRNAs greater than 3. Pathway enrichment and Gene Ontology (GO) analysis was performed with the web application Metascape using analysis including “GO Molecular Function”, “GO Biological Processes”, and “KEGG Pathway” with the default parameters.^19^ Protein‐protein interaction (PPI) analysis was carried out by different protein interaction databases like BioGrid, InWeb_IM and OmniPath by using the Metascape tool. Molecular complex detection (MCODE) algorithm was used by the Metascape tool to detect molecular complexes those were the densely connected regions in protein interaction network.^19^

The data are presented as mean ± standard deviation. The significance of differences between the groups was determined by paired Student's *t* test and/or one‐way ANOVA by the GraphPad Prism 6 software, with 0.05 as the level of significance.

## 3 RESULTS

### 3.1 A genome‐wide CRISPR/Cas9‐mediated screen to identify OGDR resistance genes

**Figure 1:**
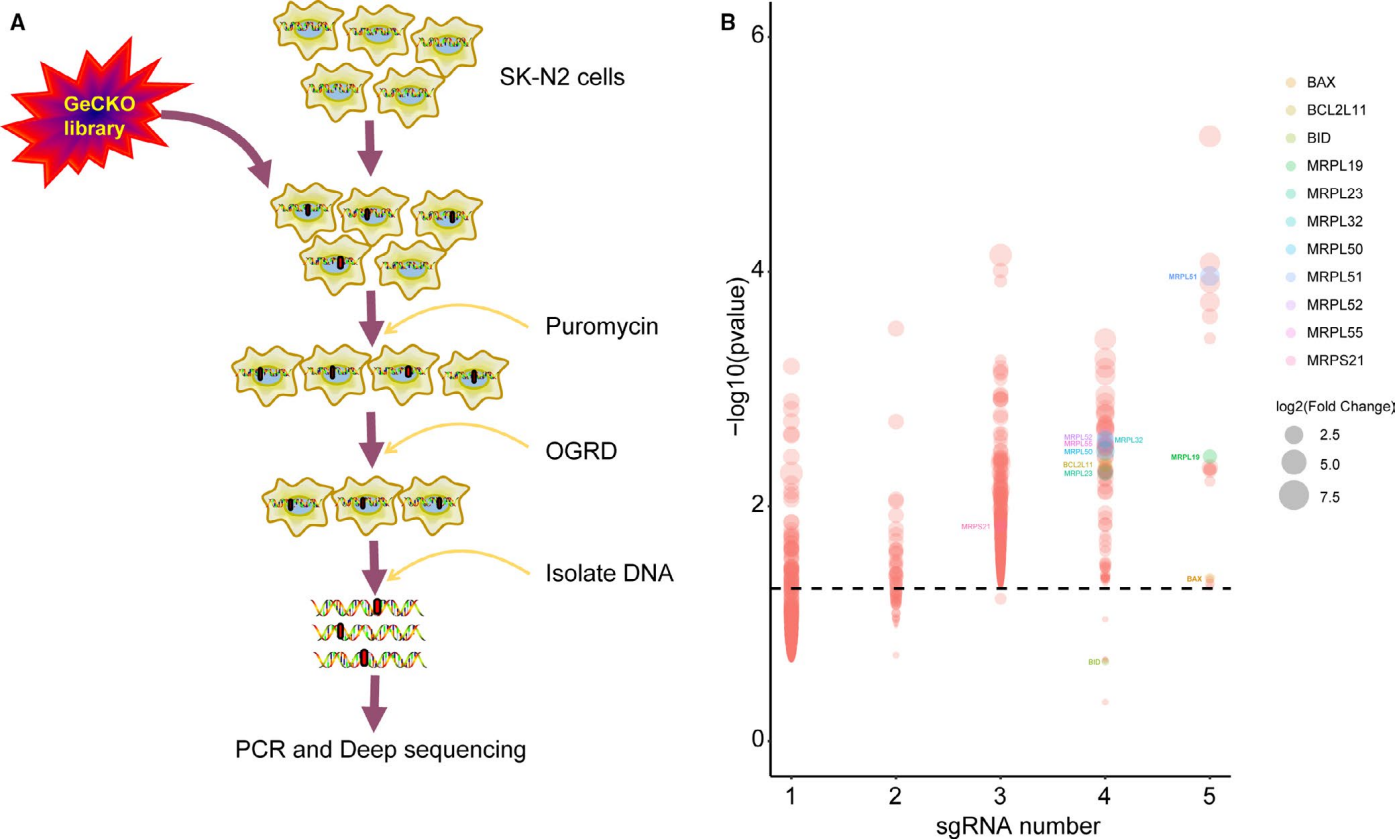
A GeCKO screen to identify genes whose loss confers OGDR resistance. A, Schematic of forward GeCKO screen in SK‐N‐BE(2) cells using pooled sgRNA libraries. B, Genes identified in the screen for oxygen‐glucose deprivation/reperfusion resistance. The *x*‐axis is the number of unique sgRNAs for each gene. The *y*‐axis (−log10 of the *P*‐value) represents the change of each sgRNA compared to control (without OGDR treatment). The size of the circle represents the fold change of reads compared to control. The genes of interest and positive control marked with colour. The line represents a corrected *P* = 0.05

The apoptosis of neurons has been widely found in ischaemia‐reperfusion injury in vitro and in vivo. The cutting‐edge CRISPR/Cas9‐mediated genome‐editing technologies provide a useful tool to discover additional modulators of ischaemia‐reperfusion signalling. We used a GeCKO lentivirus library containing 123 411 sgRNAs targeting whole 19 050 human genes and generated a pool of cells with lentivirus infection, in which every targeted gene theoretically carried a loss of function mutation in a single cell.^20^ After treating the pooled cells with OGDR insult, we collected the viable cells for analysis of the inserted sgRNAs in genome (Figure 1A). The sgRNAs amplified from the surviving cells’ genome were sequenced by next‐generation sequencing (NGS) and mapped to indexed library. The raw sequence data reported in this paper have been deposited in the Genome Sequence Archive^21^ in National Genomics Data Center,^22^ Beijing Institute of Genomics (China National Center for Bioinformation), Chinese Academy of Sciences, under accession number CRA002630 that are publicly accessible at https://bigd.big.ac.cn/gsa. The candidate genes were ranked depending on p‐values from a parametric control and the number of unique sgRNAs versus NGS reads. Enrichment of candidate sgRNAs was found in our GeCKO screening, suggesting that loss of function of these genes conferred resistance to OGDR insult. Of the 19 050 genes tested, our GeCKO screen identified BAX, BID and BCL2L11 genes that have been reported to be involved in ischaemia, hypoxia or OGDR^23^, ^24^, ^25^, ^26^ (Figure 1B and Table S1). We have also discovered that the deficiency of an interesting gene family, mitochondrial ribosomal protein (MRP) family, may contribute to protection against OGDR injury (Figure 1B).

### 3.2 Pathway and GO analysis of OGDR resistance genes and construction of PPI network

**Figure 2:**
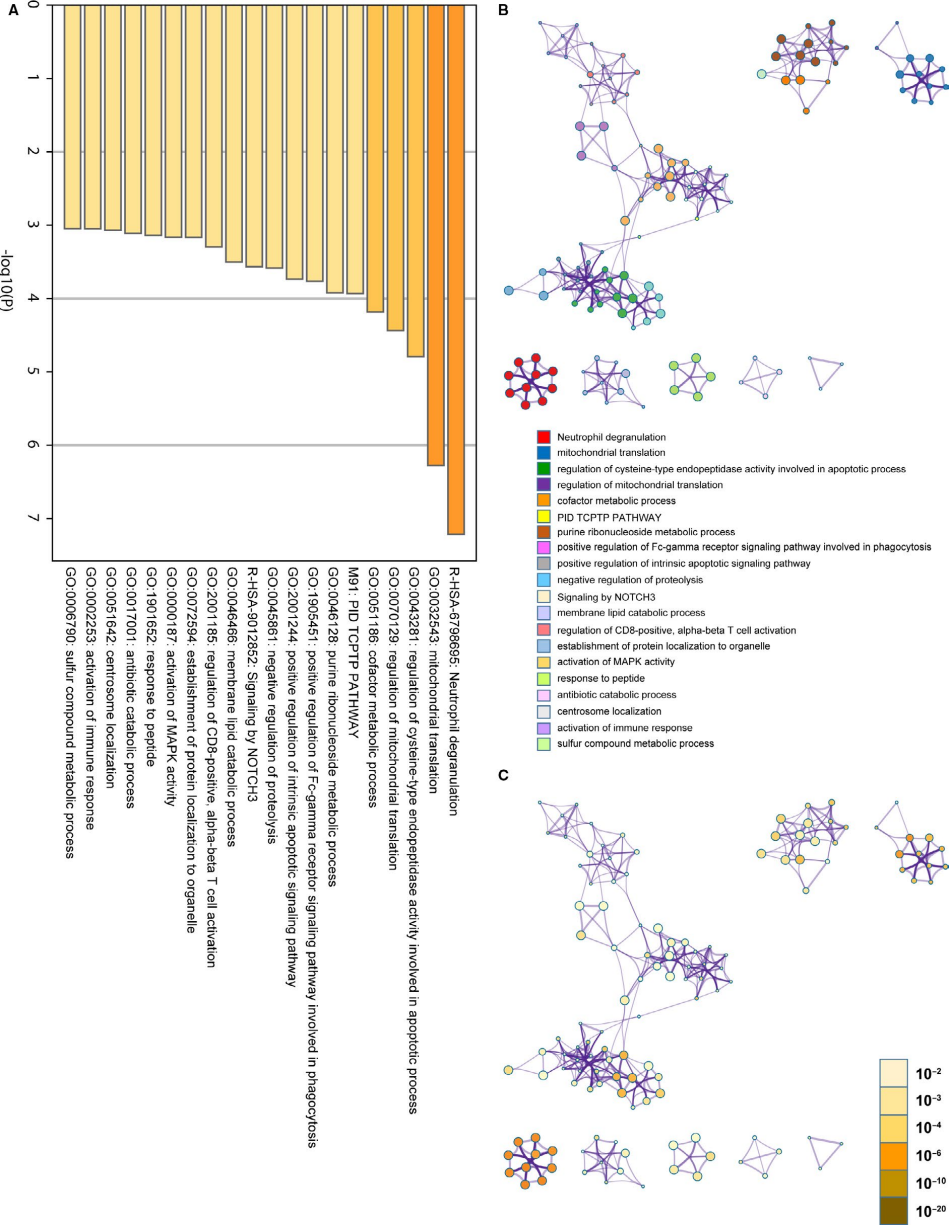
Comprehensive analysis of oxygen‐glucose deprivation/reperfusion (OGDR) resistance genes in SK‐N‐BE(2) cells by Metascape. A, The top 20 significantly enriched biological process and pathways related to OGDR resistance genes with bar graph. B, The top 20 significantly enriched biological process and pathways related to OGDR resistance genes with network. Different colours in the map represented different function groups. C, The same enrichment network has its nodes coloured by *P*‐value, as shown in the legend. The darker the colour, the more statistically significant the node is

To understand the role of these candidate genes from GeCKO screen for OGDR resistance, Metascape was used for GO and KEGG enrichment analysis of top‐rank genes with high number (3‐5) of unique sgRNAs.^19^ The top 20 significant pathways and functions were listed in accordance with p‐values (Figure 2A). The GO analysis demonstrates that OGDR resistance genes were primarily involved in neutrophil degranulation, mitochondrial translation, and regulation of cysteine‐type endopeptidase activity involved in apoptotic process and response to oxidative stress.

**Figure 3:**
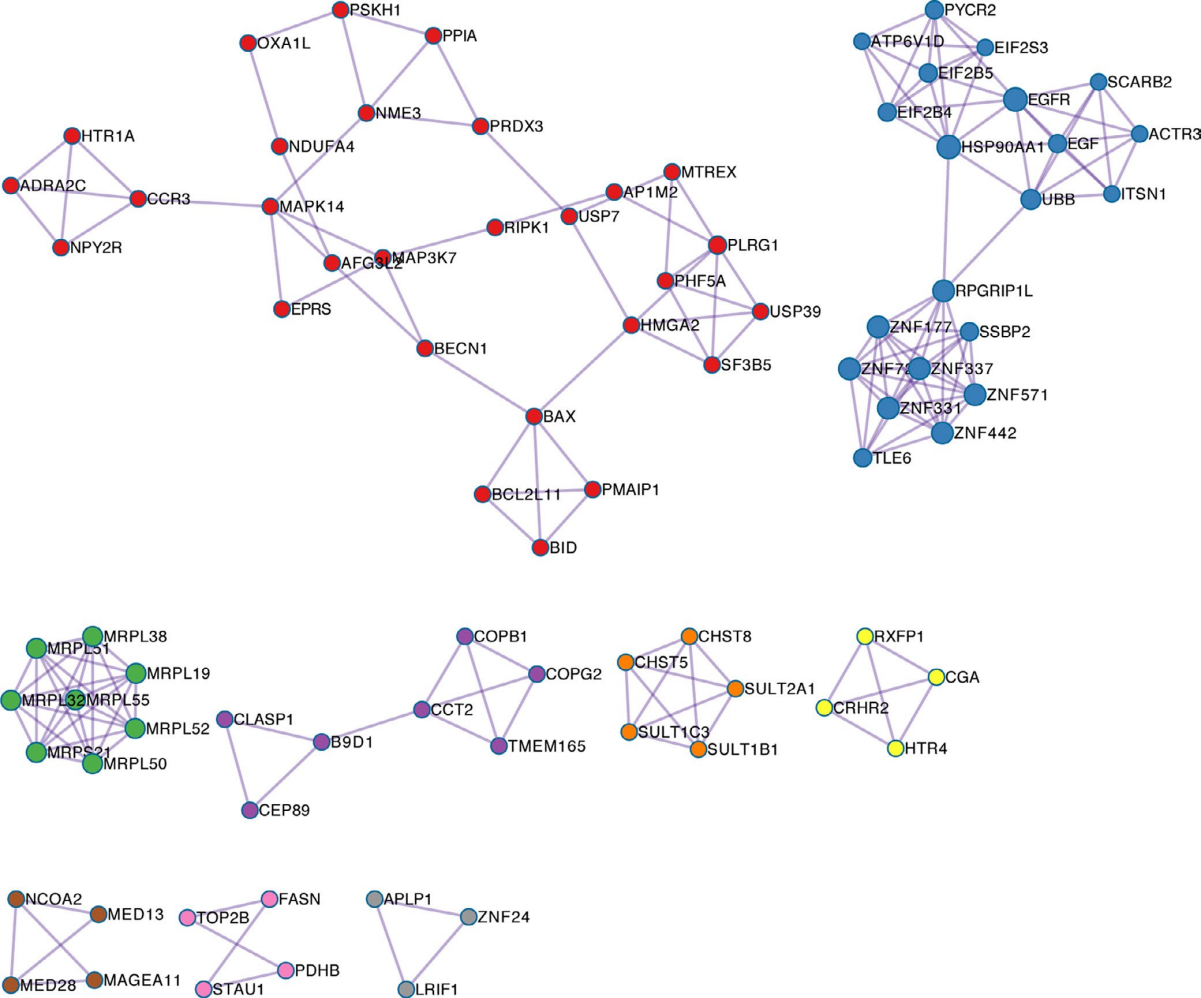
Protein‐protein interaction (PPI) network and nine most significant molecular complex detection (MCODE) components form the PPI network. MCODE algorithm was applied to this network to identify neighbourhoods where proteins are densely connected. Each node represents a protein, and the edge between nodes represents the interaction between two connected proteins

We also employed Metascape for PPI enrichment analysis to better understand the interaction among OGDR resistance genes. We used the MCODE algorithm to identify densely connected network components. A list of important genetic components in the PPI network is shown in Figure 3. The nine most important MCODE components were selected, and pathway and enrichment process analyses were independently applied to each MCODE component. The results showed that the biological functions of the MCODE components primarily included apoptosis, EGFR pathway and mitochondrial translation elongation (Table S2).

Taken together, our GeCKO screen has identified the OGDR resistance genes in SK‐N‐BE(2) cells, which were primarily involved in neutrophil degranulation, mitochondrial translation, and regulation of cysteine‐type endopeptidase activity involved in apoptotic process and response to oxidative stress. Through GO analysis, PPI analysis and MCODE algorithm, MRP‐related genes showed the most significant enrichment (Figure 2 and Table S2). Therefore, we chose these genes for subsequent verification.

### 3.3 Effect of the MRP genes from the GeCKO screen on OGDR‐induced apoptosis

**Figure 4:**
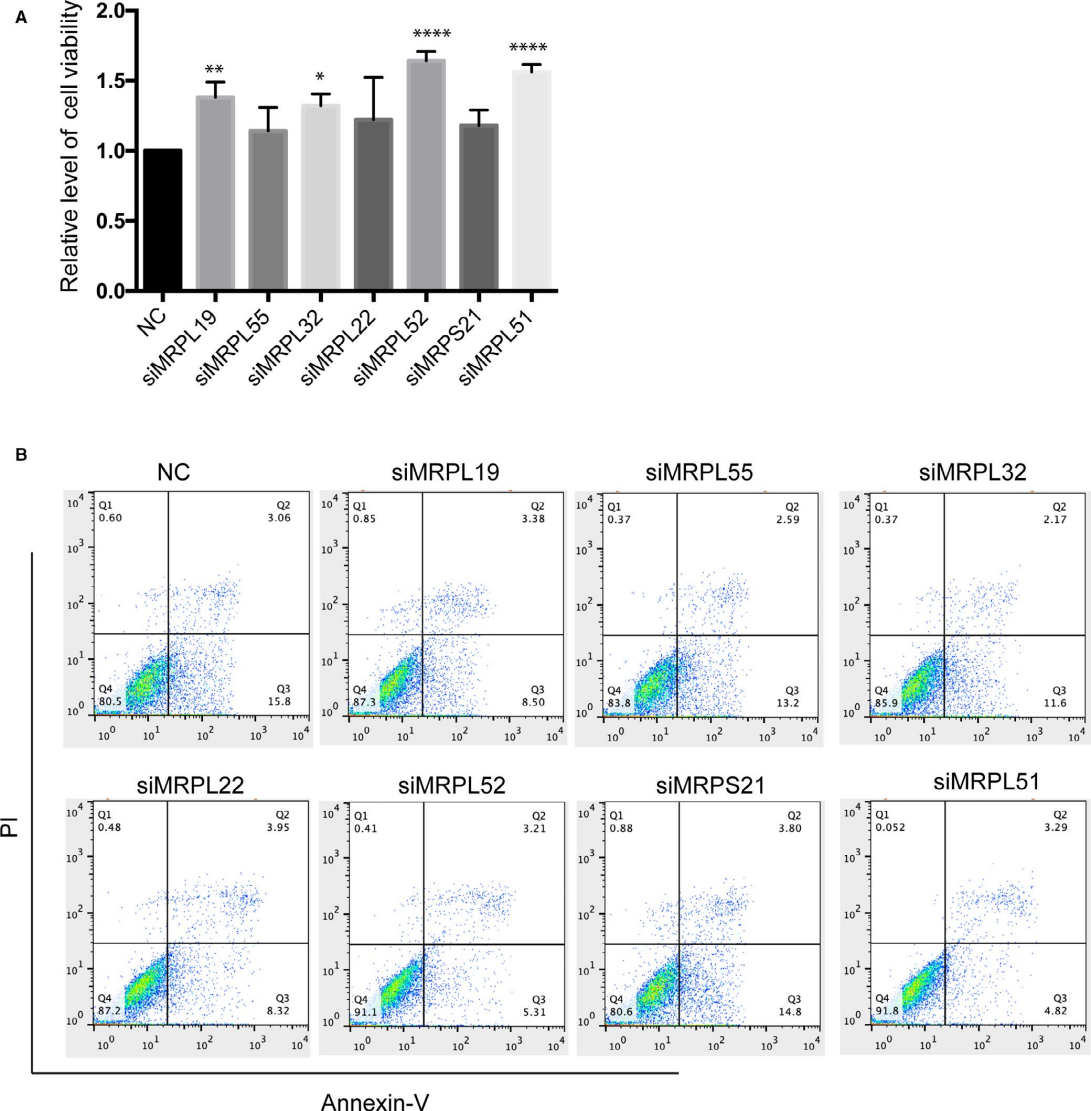
Validation of oxygen‐glucose deprivation/reperfusion (OGDR) resistance genes with increased cell viability and decreased apoptosis when knocked down. A, Confirmatory analysis of mitochondrial ribosomal protein gene family identified in the GeCKO screen. SK‐N‐BE(2) cells were transfected siRNAs targeting each candidate gene and subjected to OGDR injury. The relative levels of cell viability were analysed. B, The relative levels of apoptosis were analysed. *P < 0.05, **P < 0.01 and ****P < 0.0001

Mitochondrial DNA (mtDNA) encodes 13 proteins that are essential for the function of the oxidative phosphorylation. The mRNAs for a total of 13 proteins are translated on mitochondrial ribosomes.^27^ To validate the MRP genes from the GeCKO screen, we next knocked down these genes by expressing siRNA sequences targeting each gene. Cell viability and apoptosis of the cells transfected with siRNA and control during OGDR insult were analysed. We observed a significant increase in cell viability and a significant decrease in apoptosis during OGDR insult following knockdown of MRPL19, MRPL32, MRPL52 and MRPL51, but the effect of knockdown of MRPL55, MRPL22 and MRPS21 was not significant (Figure 4). The findings for MRPL19, MRPL32, MRPL52 and MRPL51 are consistent with the integrative analysis during the genome‐wide CRISPR/Cas9‐mediated screen, which suggests that these genes may sensitize cells to apoptosis following OGDR injury.

### 3.4 Expression pattern of MRPL19 and MRPL51 protein in SK‐N‐BE(2) cells upon OGDR insult

**Figure 5:**
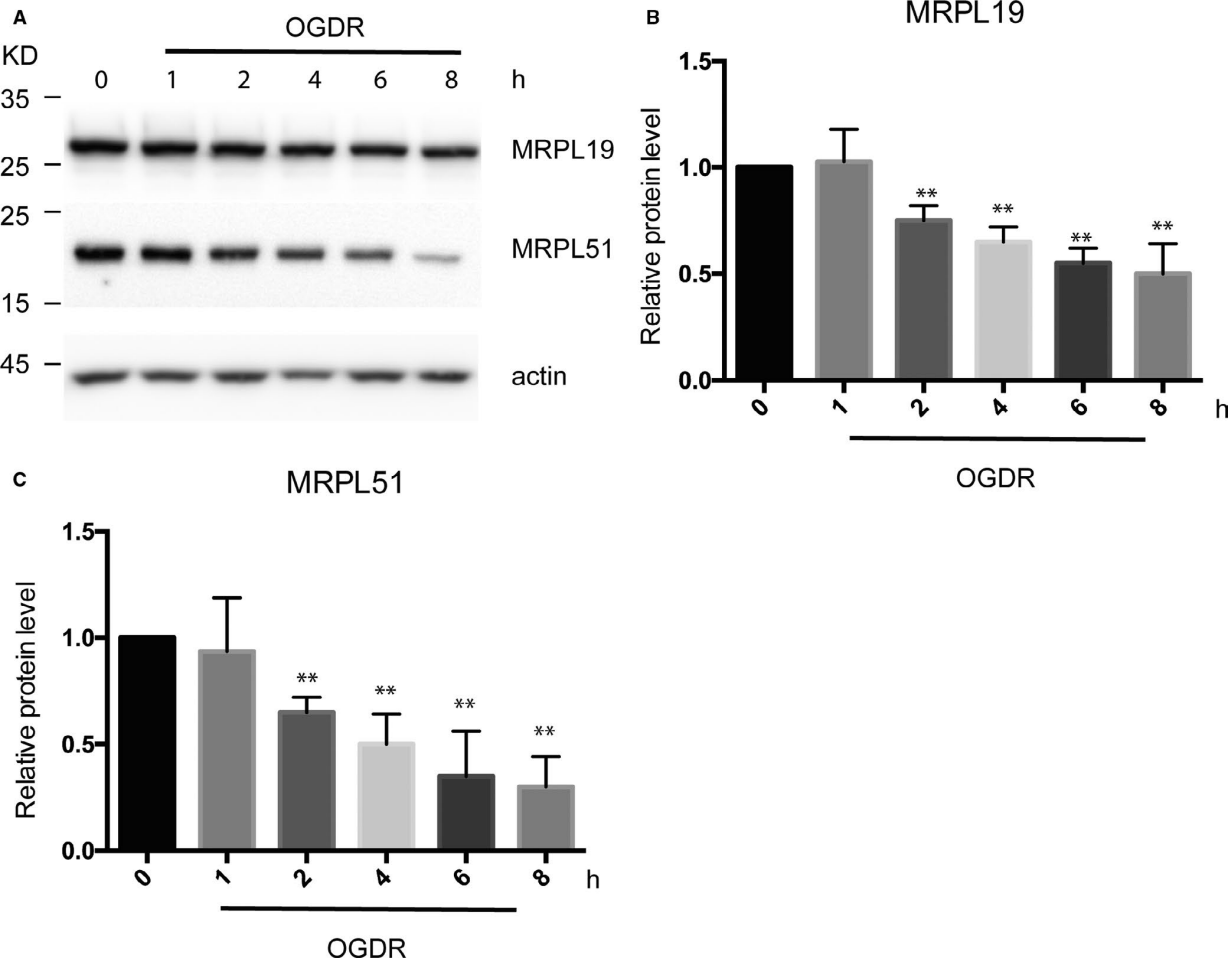
Oxygen‐glucose deprivation/reperfusion (OGDR) affects the protein level of MRPL19 and MRPL51 in SK‐N‐BE(2) cells. A, Western blot analysis of MRPL19 and MRPL51 expression in SK‐N‐BE(2) cells upon OGDR insult. Actin was used as a loading control. B, Quantitative analysis of MRPL19 expression in SK‐N‐BE(2) cells upon OGDR insult. C, Quantitative analysis of MRPL51 expression in SK‐N‐BE(2) cells upon OGDR insult. Data are presented as the mean ± SD. Asterisks indicate statistically significant difference compared with control. **P < 0.01

We further determined the expression profile of MRPL19 and MRPL51 protein at different reperfusion time points following 4 hours OGD. Western blot analysis showed similar expression of MRPL19 and MRPL51 between SK‐N‐BE(2) cells without OGDR treatment and SK‐N‐BE(2) cells subjected to 1‐hour reperfusion following 4‐hour OGD. However, expression of MRPL19 and MRPL51 was strongly decreased in SK‐N‐BE(2) cells after 2‐ to 8‐hour reperfusion following 4‐hour OGD (Figure 5). This suggests that MRPL protein may play a protective role or promote apoptosis during OGDR.

## 4 DISCUSSION

Cerebral ischaemia‐reperfusion injury aggravates neurological damage and brain dysfunction in ischaemic stroke. The underlying mechanisms of ischaemia‐reperfusion injury are complicated, and the precise aetiology and origin are still largely unrevealed. In this study, we performed a whole genome‐scale CRISPR‐Cas9 loss of function selection screen in SK‐N‐BE(2) cells to discover potential contributors to ischaemia‐reperfusion resistance. Our results suggest that neutrophil degranulation, mitochondrial translation, and regulation of cysteine‐type endopeptidase activity involved in apoptotic process and response to oxidative stress were involved in OGDR resistance. As we know, ischaemia‐reperfusion insult attracts neutrophils to the ischaemic brain. After ischaemia‐reperfusion injury, neutrophils accumulate in the perivascular spaces and leptomeninges, and even in the infarcted brain parenchyma.^28^ Neutrophil PKC delta plays a critical role in cerebral ischaemia‐reperfusion injury. PKC delta inhibitors could confer neuroprotective effect against ischaemia‐reperfusion injury.^29^ Mitochondrial cardiolipin oxidative signalling dysfunction also plays an important role in the development of cerebral ischaemia‐reperfusion injury.^30^ Therefore, the candidate genes analysed by Metascape in our study may be of great importance in cerebral ischaemia‐reperfusion injury.

Currently, the major limitation of cerebral ischaemia‐reperfusion management is the lack of clinically effective therapeutic interventions. As we know, mitochondrion is the key organelle for ATP production and also plays a critical role in apoptosis, oxidative stress and mitophagy. It is also important in cerebral ischaemia‐reperfusion injury. Cerebral ischaemia‐reperfusion injury‐induced brain damage is related to multiple independently fatal terminal pathways in the mitochondria. Mitochondria have been proved to be a promising therapeutic target for neuroprotection against ischaemia‐reperfusion injury.^31^, ^32^ In this study, we found that inhibition of MRPL19, MRPL32, MRPL52 and MRPL51 increased cell viability and decreased apoptosis after OGDR treatment, suggesting that these MRPs are potential therapeutic targets.

Mammalian mitochondrial ribosomes (mitoribosomes) are highly divergent. They synthesize 13 proteins encoded by the mitochondrial genome that are essential for oxidative phosphorylation.^33^ The MRPs are involved in many crucial cellular processes, such as mitochondrial homeostasis regulation, cell cycle regulation and apoptosis.^34^ There has been an increasing body of literature describing an alternative role for several MRPs as apoptosis‐inducing factors.^33^, ^35^, ^36^ MRPL41 enhances p53 stability, regulates p53 translocation to the mitochondria and contributes to p53‐induced apoptosis in response to growth‐inhibitory conditions.^37^ Death‐associated protein 3 (DAP3) and the programmed cell death protein 9 (PCDP9) are components of the mitochondrial ribosome and serve as a major component in cellular apoptotic signalling pathways.^38^, ^39^ MRPL65, a homology to the chicken pro‐apoptotic protein p52, activates JNK1 pathway and induces apoptosis.^40^ Hypoxia strongly decreases the expression of MRPs.^41^ In this study, we found that OGDR decreased MRPL19 and MRPL51 expression. The decreased expression of MRPs during OGDR would reduce mitochondrial protein synthesis and inhibit the activity of the respiratory chain, thereby reducing the production of ROS and protecting against OGDR injury. Meanwhile, as apoptosis‐inducing factors, the decreased MRPs would also inhibit OGDR‐induced apoptosis. Therefore, future therapeutic interventions targeting MRPL19 and MRPL51 may be exploited for protecting against ischaemia‐reperfusion brain injury.

In summary, we have successfully applied a genome‐scale CRISPR‐Cas9 screen in SK‐N‐BE(2) cells to identify OGDR resistance genes. We have identified a class of genes contributed to OGDR resistance, including genes involved in neutrophil degranulation, mitochondrial translation, regulation of cysteine‐type endopeptidase activity involved in apoptotic process and response to oxidative stress. MRPL19 and MRPL51 knockdown decreased OGDR‐induced apoptosis, while OGDR treatment down‐regulated the protein expression of MRPL19 and MRPL51. Further identification and analysis of the genes identified by this genome‐scale CRISPR‐Cas9 screen will provide more understanding of the pathogenic mechanisms underlying ischaemia‐reperfusion insult and provide new therapeutic targets for cerebral ischaemia‐reperfusion injury.

## CONFLICT OF INTEREST

The author(s) confirm that this article content has no conflicts of interest.

## AUTHOR CONTRIBUTIONS

**Xinjie Guan:** Investigation (equal); methodology (equal). **Hainan Zhang:** Investigation (equal); methodology (equal). **Haiyun Qin:** Data curation (equal); formal analysis (equal). **Chunli Chen:** Formal analysis (equal); validation (equal). **Zhiping Hu:** Conceptualization (equal); supervision (equal). **Jieqiong Tan:** Conceptualization (equal); investigation (equal); writing‐original draft (equal); writing‐review & editing (equal). **Liuwang Zeng:** Conceptualization (equal); funding acquisition (equal); methodology (equal); supervision (equal); writing‐original draft (equal); writing‐review & editing (equal).

## Supporting information

Supplementary Material

Supplementary Material

## Data Availability

The raw sequence data reported in this paper have been deposited in the Genome Sequence Archive in National Genomics Data Center, Beijing Institute of Genomics (China National Center for Bioinformation), Chinese Academy of Sciences, under accession number CRA002630 that are publicly accessible at https://bigd.big.ac.cn/gsa.
