# Supplementary material for: CRISPR/Cas9‐mediated whole genomic wide knockout screening identifies mitochondrial ribosomal proteins involving in oxygen‐glucose deprivation/reperfusion resistance
Source: J Cell Mol Med. 2020 Jul 2;24(16):9313–22. doi: 10.1111/jcmm.15580 (PMC7417733; doi:10.1111/jcmm.15580)
Supplement: Supplementary file 1 — Supplementary Material [file JCMM-24-9313-s001.pdf]

Table S1 Genes identified in the screening for OGDR resistance

Gene	Sumary	unique gene	sgRNA ID, reads
CASK	7394	5	HGLibB_07281,3779|HGLibA_07286,2603|HGLibA_07285,80|HGLibB_07280,1|HGLibA_07287,931|HGLibB_07279,0
NEU2	5685	5	HGLibA_31713,653|HGLibB_31669,1720|HGLibA_31714,872|HGLibB_31671,1127|HGLibA_31712,1313|HGLibB_31670,0
MRPL51	4687	5	HGLibA_30011,3295|HGLibB_29970,57|HGLibB_29969,335|HGLibA_30012,200|HGLibB_29971,800|HGLibA_30010,0
NADK	4251	5	HGLibA_31046,1353|HGLibB_31002,79|HGLibB_31003,25|HGLibA_31044,2782|HGLibB_31004,12|HGLibA_31045,0
MTRNR2L4	4149	5	HGLibB_30439,1267|HGLibB_30440,2150|HGLibA_30481,3|HGLibB_30438,1|HGLibA_30480,728|HGLibA_30482,0
ZNF442	3921	5	HGLibB_55941,1236|HGLibB_55940,1699|HGLibA_56009,3|HGLibB_55942,2|HGLibA_56007,981|HGLibA_56008,0
TPD52	3362	5	HGLibA_51111,564|HGLibA_51112,279|HGLibB_51047,1221|HGLibB_51048,1253|HGLibA_51110,45|HGLibB_51049,0
XKRY	3258	5	HGLibA_54541,82|HGLibA_54540,217|HGLibA_54542,501|HGLibB_54475,1851|HGLibB_54474,607
EPPIN	2661	5	HGLibB_15335,83|HGLibA_15356,4|HGLibA_15355,1|HGLibB_15333,1369|HGLibA_15357,1204|HGLibB_15334,0
MAP3K7	2431	5	HGLibB_28154,1177|HGLibA_28197,1214|HGLibA_28196,1|HGLibB_28156,9|HGLibA_28195,30|HGLibB_28155,0
MAPK14	2395	5	HGLibB_28222,1092|HGLibB_28220,1297|HGLibB_28221,3|HGLibA_28263,2|HGLibA_28261,1|HGLibA_28262,0
MRPL19	1726	5	HGLibB_29878,1236|HGLibB_29876,3|HGLibA_29917,447|HGLibA_29919,19|HGLibA_29918,21|HGLibB_29877,0
BAX	1385	5	HGLibA_04181,385|HGLibA_04183,10|HGLibB_04179,20|HGLibA_04182,968|HGLibB_04181,2|HGLibB_04180,0
EIF2B4	1173	5	HGLibA_14743,1000|HGLibB_14722,33|HGLibB_14721,70|HGLibB_14723,62|HGLibA_14744,8|HGLibA_14745,0
STAT1	1018	5	HGLibB_47325,6|HGLibB_47324,3|HGLibA_47381,1000|HGLibB_47323,8|HGLibA_47382,1|HGLibA_47380,0
EIF2B5	344	5	HGLibB_14724,22|HGLibA_14746,300|HGLibB_14725,4|HGLibA_14748,8|HGLibB_14726,10|HGLibA_14747,0
EIF2S3	72	5	HGLibB_14736,3|HGLibA_14758,40|HGLibA_14760,9|HGLibB_14737,10|HGLibA_14759,10|HGLibB_14738,0
ZNF697	6696	4	HGLibA_56564,24|HGLibA_56563,2077|HGLibA_56562,3961|HGLibB_56497,634|HGLibB_56496,0|HGLibB_56495,0
WFDC8	6607	4	HGLibA_54273,1268|HGLibB_54209,600|HGLibA_54274,4554|HGLibB_54208,185|HGLibB_54207,0|HGLibA_54275,0
IMMT	5211	4	HGLibA_23236,478|HGLibB_23202,2027|HGLibB_23203,1299|HGLibB_23204,1407|HGLibA_23235,0|HGLibA_23234,0
MTRR	4883	4	HGLibA_30494,1108|HGLibB_30450,1627|HGLibB_30452,1518|HGLibA_30492,630|HGLibA_30493,0|HGLibB_30451,0
SLC9A2	4863	4	HGLibA_45404,1176|HGLibB_45350,2352|HGLibA_45402,1|HGLibA_45403,1334|HGLibB_45351,0|HGLibB_45349,0
LMAN2	4736	4	HGLibA_26663,2856|HGLibB_26625,2|HGLibA_26664,625|HGLibA_26662,1253|HGLibB_26626,0|HGLibB_26627,0
KRTAP5-10	4426	4	HGLibA_25786,1373|HGLibB_25748,2859|HGLibA_25784,186|HGLibA_25785,8|HGLibB_25749,0|HGLibB_25750,0
MRPL50	4323	4	HGLibB_29966,24|HGLibA_30007,1299|HGLibB_29968,2001|HGLibA_30009,999|HGLibA_30008,0|HGLibB_29967,0
CADM1	4233	4	HGLibA_06965,1205|HGLibB_06961,1672|HGLibA_06966,1343|HGLibB_06959,13|HGLibB_06960,0|HGLibA_06964,0
CREBL2	4026	4	HGLibA_11111,1202|HGLibA_11112,1377|HGLibA_11110,1378|HGLibB_11100,69|HGLibB_11098,0|HGLibB_11099,0
APOL3	3992	4	HGLibA_02532,704|HGLibB_02531,917|HGLibA_02534,1117|HGLibA_02533,1254|HGLibB_02530,0|HGLibB_02532,0
MRPL52	3832	4	HGLibA_30013,3603|HGLibA_30014,129|HGLibB_29974,45|HGLibB_29972,55|HGLibA_30015,0|HGLibB_29973,0
CTXN2	3785	4	HGLibB_11746,1282|HGLibA_11759,2479|HGLibA_11758,22|HGLibB_11745,2|HGLibA_11757,0|HGLibB_11747,0
PANK3	3546	4	HGLibA_35102,470|HGLibB_35057,1323|HGLibA_35104,1432|HGLibB_35058,321|HGLibA_35103,0|HGLibB_35056,0
ZBTB2	3354	4	HGLibB_54768,1411|HGLibB_54769,372|HGLibB_54767,1220|HGLibA_54834,351|HGLibA_54835,0|HGLibA_54836,0
OR52M1	3277	4	HGLibB_34145,1231|HGLibA_34191,6|HGLibB_34144,1340|HGLibA_34189,700|HGLibB_34146,0|HGLibA_34190,0
OR6C75	3188	4	HGLibA_34408,619|HGLibB_34365,1211|HGLibA_34409,2|HGLibB_34364,1356|HGLibA_34410,0|HGLibB_34363,0
IPO11	3142	4	HGLibB_23428,1418|HGLibB_23426,542|HGLibB_23427,832|HGLibA_23460,350|HGLibA_23461,0|HGLibA_23459,0
KDM4D	3130	4	HGLibB_24480,715|HGLibB_24482,1052|HGLibB_24481,34|HGLibA_24515,1329|HGLibA_24516,0|HGLibA_24514,0
ZNF781	3120	4	HGLibB_56671,1599|HGLibB_56670,1318|HGLibA_56738,19|HGLibB_56669,184|HGLibA_56736,0|HGLibA_56737,0
PLRG1	3118	4	HGLibA_37222,1317|HGLibA_37223,630|HGLibB_37174,1166|HGLibA_37221,5|HGLibB_37173,0|HGLibB_37172,0
HAO2	3022	4	HGLibB_20806,73|HGLibA_20834,259|HGLibB_20808,1269|HGLibA_20835,1421|HGLibB_20807,0|HGLibA_20836,0
SLC25A37	2952	4	HGLibA_44723,647|HGLibB_44670,33|HGLibB_44671,308|HGLibB_44669,1964|HGLibA_44724,0|HGLibA_44722,0
ADAMTS13	2892	4	HGLibA_00801,657|HGLibA_00799,2226|HGLibA_00800,3|HGLibB_00797,6|HGLibB_00798,0|HGLibB_00799,0
PABPN1L	2871	4	HGLibB_34900,681|HGLibB_34899,719|HGLibA_34946,10|HGLibB_34898,1461|HGLibA_34945,0|HGLibA_34944,0
SPEF2	2851	4	HGLibB_46557,1509|HGLibA_46611,690|HGLibA_46612,3|HGLibB_46556,649|HGLibB_46555,0|HGLibA_46613,0
ANAPC7	2781	4	HGLibA_01888,175|HGLibA_01889,18|HGLibB_01887,1294|HGLibB_01888,1294|HGLibB_01886,0|HGLibA_01890,0
WDR16	2742	4	HGLibB_53967,1280|HGLibA_54034,69|HGLibB_53969,284|HGLibA_54033,1109|HGLibA_54035,0|HGLibB_53968,0
GINS4	2525	4	HGLibB_19087,1342|HGLibB_19088,1173|HGLibA_19113,2|HGLibB_19086,8|HGLibA_19112,0|HGLibA_19111,0
DNMT3B	2514	4	HGLibA_13703,1227|HGLibA_13704,1265|HGLibB_13684,11|HGLibB_13685,11|HGLibB_13683,0|HGLibA_13705,0
KRTAP20-3	2466	4	HGLibA_25706,179|HGLibB_25669,821|HGLibA_25704,1125|HGLibB_25670,341|HGLibA_25705,0|HGLibB_25668,0
ZC2HC1C	2459	4	HGLibB_54875,70|HGLibB_54877,1374|HGLibA_54942,867|HGLibB_54876,148|HGLibA_54943,0|HGLibA_54944,0
DNAI1	2349	4	HGLibA_13504,160|HGLibB_13485,1159|HGLibA_13503,685|HGLibB_13484,345|HGLibB_13486,0|HGLibA_13505,0
HLA-DQB2	2314	4	HGLibA_21630,1345|HGLibA_21629,957|HGLibB_21601,11|HGLibA_21631,1|HGLibB_21602,0|HGLibB_21603,0
DOK4	2291	4	HGLibA_13776,1397|HGLibB_13756,114|HGLibB_13755,730|HGLibA_13775,50|HGLibB_13754,0|HGLibA_13777,0
KRTAP13-4	2236	4	HGLibB_25615,1318|HGLibB_25616,250|HGLibA_25650,656|HGLibA_25651,12|HGLibA_25652,0|HGLibB_25614,0
MIPEP	2201	4	HGLibB_29254,1421|HGLibB_29252,7|HGLibA_29295,687|HGLibA_29293,86|HGLibB_29253,0|HGLibA_29294,0
GDPD2	2117	4	HGLibA_18894,1|HGLibA_18893,1354|HGLibB_18868,429|HGLibA_18892,333|HGLibB_18869,0|HGLibB_18867,0
CNOT1	2109	4	HGLibB_10316,1383|HGLibB_10315,27|HGLibA_10326,581|HGLibB_10314,118|HGLibA_10327,0|HGLibA_10325,0
RNASE7	2101	4	HGLibB_41364,2|HGLibA_41414,358|HGLibB_41363,1732|HGLibB_41362,9|HGLibA_41415,0|HGLibA_41416,0
NT5E	2048	4	HGLibB_32785,181|HGLibB_32784,599|HGLibA_32828,11|HGLibA_32830,1257|HGLibA_32829,0|HGLibB_32786,0
PRDX3	1992	4	HGLibB_38364,1988|HGLibA_38414,2|HGLibB_38363,1|HGLibA_38412,1|HGLibB_38365,0|HGLibA_38413,0
HSFY1	1969	4	HGLibA_22242,300|HGLibA_22240,361|HGLibB_22213,1306|HGLibA_22241,2|HGLibB_22212,0
UQCC2	1923	4	HGLibA_53102,2|HGLibB_53037,1338|HGLibB_53036,212|HGLibA_53101,371|HGLibB_53035,0
ACER2	1923	4	HGLibB_00431,1178|HGLibB_00432,743|HGLibA_00431,1|HGLibB_00430,1|HGLibA_00430,0|HGLibA_00432,0
EIF2A	1922	4	HGLibA_14720,922|HGLibB_14698,200|HGLibB_14699,30|HGLibA_14719,770|HGLibB_14697,0|HGLibA_14721,0
FCRLA	1887	4	HGLibB_17221,6|HGLibB_17220,1824|HGLibA_17243,56|HGLibA_17244,1|HGLibB_17219,0|HGLibA_17242,0
APOL4	1805	4	HGLibB_02535,184|HGLibA_02535,1511|HGLibA_02537,1|HGLibB_02533,109|HGLibB_02534,0|HGLibA_02536,0
VSIG2	1751	4	HGLibB_53779,321|HGLibA_53844,100|HGLibB_53780,777|HGLibA_53846,553|HGLibA_53845,0|HGLibB_53778,0
PRAMEF12	1740	4	HGLibB_38238,1574|HGLibB_38237,1|HGLibB_38239,154|HGLibA_38288,11|HGLibA_38287,0|HGLibA_38286,0
BACE1	1625	4	HGLibA_04059,1129|HGLibB_04056,168|HGLibB_04057,195|HGLibB_04058,133|HGLibA_04058,0|HGLibA_04060,0
KRT86	1597	4	HGLibA_25574,559|HGLibA_25572,364|HGLibB_25538,59|HGLibB_25536,615|HGLibA_25573,0|HGLibB_25537,0
ARL11	1563	4	HGLibB_02924,8|HGLibB_02925,1380|HGLibA_02926,92|HGLibB_02926,83|HGLibA_02927,0|HGLibA_02928,0
MRPL55	1561	4	HGLibB_29982,188|HGLibB_29981,1000|HGLibB_29983,36|HGLibA_30023,337|HGLibA_30022,0|HGLibA_30024,0
CMAS	1528	4	HGLibB_10168,1346|HGLibA_10178,10|HGLibB_10170,170|HGLibA_10180,2|HGLibB_10169,0|HGLibA_10179,0
MSI1	1499	4	HGLibB_30163,1355|HGLibA_30206,4|HGLibA_30205,27|HGLibB_30165,113|HGLibA_30204,0|HGLibB_30164,0
BCL2L11	1259	4	HGLibA_04308,1009|HGLibA_04307,12|HGLibB_04306,200|HGLibB_04307,38|HGLibA_04309,0|HGLibB_04305,0

<table>
<tr><td>HNF1B</td><td>1242</td><td>4</td><td>HGLibA_21778,1217|HGLibA_21776,10|HGLibB_21748,2|HGLibB_21749,13|HGLibB_21747,0|HGLibA_21777,0</td></tr>
<tr><td>KLRC2</td><td>1175</td><td>4</td><td>HGLibB_25254,959|HGLibA_25287,156|HGLibB_25252,7|HGLibB_25253,53|HGLibA_25289,0|HGLibA_25288,0</td></tr>
<tr><td>ESD</td><td>1145</td><td>4</td><td>HGLibB_15541,1|HGLibB_15542,977|HGLibB_15540,82|HGLibA_15562,85|HGLibA_15564,0|HGLibA_15563,0</td></tr>
<tr><td>CLIP4</td><td>1021</td><td>4</td><td>HGLibA_10050,659|HGLibA_10051,333|HGLibB_10042,2|HGLibA_10052,27|HGLibB_10041,0|HGLibB_10043,0</td></tr>
<tr><td>MAPKAPK5</td><td>1008</td><td>4</td><td>HGLibB_28266,34|HGLibB_28265,358|HGLibA_28307,42|HGLibB_28267,574|HGLibA_28306,0|HGLibA_28308,0</td></tr>
<tr><td>MRPL23</td><td>852</td><td>4</td><td>HGLibA_29934,445|HGLibB_29891,7|HGLibB_29893,234|HGLibA_29933,166|HGLibB_29892,0|HGLibA_29932,0</td></tr>
<tr><td>CDC20B</td><td>808</td><td>4</td><td>HGLibA_08520,1|HGLibA_08522,3|HGLibB_08514,315|HGLibB_08515,489|HGLibB_08513,0|HGLibA_08521,0</td></tr>
<tr><td>SLC10A4</td><td>786</td><td>4</td><td>HGLibB_44285,83|HGLibB_44287,29|HGLibA_44340,305|HGLibA_44338,369|HGLibA_44339,0|HGLibB_44286,0</td></tr>
<tr><td>PSKH1</td><td>768</td><td>4</td><td>HGLibA_39063,23|HGLibB_39016,38|HGLibB_39015,2|HGLibA_39064,705|HGLibA_39065,0|HGLibB_39014,0</td></tr>
<tr><td>LGI4</td><td>621</td><td>4</td><td>HGLibA_26403,2|HGLibA_26402,5|HGLibB_26364,2|HGLibB_26366,612|HGLibB_26365,0|HGLibA_26401,0</td></tr>
<tr><td>MRPL32</td><td>608</td><td>4</td><td>HGLibB_29909,41|HGLibA_29952,167|HGLibB_29911,301|HGLibA_29951,99|HGLibB_29910,0|HGLibA_29950,0</td></tr>
<tr><td>BID</td><td>469</td><td>4</td><td>HGLibB_04498,460|HGLibA_04500,3|HGLibB_04499,2|HGLibA_04499,4|HGLibA_04501,0|HGLibB_04497,0</td></tr>
<tr><td>RECQL</td><td>467</td><td>4</td><td>HGLibB_40716,8|HGLibB_40717,249|HGLibB_40715,186|HGLibA_40767,24|HGLibA_40766,0|HGLibA_40768,0</td></tr>
<tr><td>ZNF729</td><td>465</td><td>4</td><td>HGLibB_56575,3|HGLibB_56573,367|HGLibA_56641,31|HGLibA_56642,64|HGLibA_56640,0|HGLibB_56574,0</td></tr>
<tr><td>ATP10D</td><td>397</td><td>4</td><td>HGLibA_03550,2|HGLibB_03550,2|HGLibB_03549,3|HGLibA_03552,390|HGLibB_03548,0|HGLibA_03551,0</td></tr>
<tr><td>PNKP</td><td>229</td><td>4</td><td>HGLibA_37350,1|HGLibA_37351,23|HGLibA_37352,2|HGLibB_37301,203|HGLibB_37303,0|HGLibB_37302,0</td></tr>
<tr><td>KIAA1841</td><td>98</td><td>4</td><td>HGLibA_24822,1|HGLibB_24790,1|HGLibB_24789,83|HGLibA_24824,13|HGLibB_24788,0|HGLibA_24823,0</td></tr>
<tr><td>SIGLEC5</td><td>96</td><td>4</td><td>HGLibB_44087,92|HGLibA_44140,1|HGLibB_44088,2|HGLibB_44089,1|HGLibA_44141,0|HGLibA_44142,0</td></tr>
<tr><td>COQ5</td><td>74</td><td>4</td><td>HGLibB_10746,14|HGLibA_10759,43|HGLibA_10757,15|HGLibB_10747,2|HGLibA_10758,0|HGLibB_10748,0</td></tr>
<tr><td>SULT1B1</td><td>20</td><td>4</td><td>HGLibB_47687,2|HGLibA_47743,3|HGLibB_47685,13|HGLibA_47744,2|HGLibB_47686,0|HGLibA_47742,0</td></tr>
<tr><td>USP17L20</td><td>7691</td><td>3</td><td>HGLibA_53244,1522|HGLibA_53246,5985|HGLibA_53245,184|HGLibB_53178,0</td></tr>
<tr><td>C7orf49</td><td>6409</td><td>3</td><td>HGLibB_06543,2492|HGLibB_06544,2687|HGLibB_06545,1230|HGLibA_06548,0|HGLibA_06547,0|HGLibA_06549,0</td></tr>
<tr><td>RCBTB2</td><td>5751</td><td>3</td><td>HGLibA_40681,80|HGLibB_40630,3407|HGLibB_40628,2264|HGLibA_40679,0|HGLibB_40629,0|HGLibA_40680,0</td></tr>
<tr><td>HSP90AA1</td><td>5452</td><td>3</td><td>HGLibA_22250,2560|HGLibB_22220,1477|HGLibA_22249,1415|HGLibA_22251,0|HGLibB_22222,0|HGLibB_22221,0</td></tr>
<tr><td>MFSD3</td><td>5410</td><td>3</td><td>HGLibA_29098,1100|HGLibB_29058,1913|HGLibA_29099,2397|HGLibA_29100,0|HGLibB_29059,0|HGLibB_29057,0</td></tr>
<tr><td>MLKL</td><td>5146</td><td>3</td><td>HGLibB_29349,1010|HGLibA_29389,1379|HGLibA_29390,2757|HGLibA_29391,0|HGLibB_29350,0|HGLibB_29348,0</td></tr>
<tr><td>CD99_X</td><td>5120</td><td>3</td><td>HGLibB_08482,3703|HGLibA_08488,1415|HGLibB_08483,2|HGLibA_08489,0|HGLibB_08481,0|HGLibA_08490,0</td></tr>
<tr><td>HFE</td><td>5040</td><td>3</td><td>HGLibB_21196,4342|HGLibB_21198,697|HGLibB_21197,1|HGLibA_21224,0|HGLibA_21225,0|HGLibA_21226,0</td></tr>
<tr><td>FAM221A</td><td>4820</td><td>3</td><td>HGLibB_16494,1868|HGLibA_16515,1|HGLibA_16516,2951|HGLibB_16493,0|HGLibA_16517,0|HGLibB_16495,0</td></tr>
<tr><td>LYZ</td><td>4780</td><td>3</td><td>HGLibA_27782,2551|HGLibB_27740,2041|HGLibA_27783,188|HGLibB_27742,0|HGLibB_27741,0|HGLibA_27781,0</td></tr>
<tr><td>IL13RA1</td><td>4687</td><td>3</td><td>HGLibB_22944,1054|HGLibB_22943,3631|HGLibB_22942,2|HGLibA_22974,0|HGLibA_22975,0|HGLibA_22973,0</td></tr>
<tr><td>DENND4A</td><td>4661</td><td>3</td><td>HGLibB_12931,1295|HGLibB_12932,1283|HGLibA_12951,2083|HGLibB_12933,0|HGLibA_12950,0|HGLibA_12949,0</td></tr>
<tr><td>UNC119B</td><td>4555</td><td>3</td><td>HGLibA_52989,697|HGLibA_52990,3281|HGLibB_52926,577|HGLibB_52924,0|HGLibB_52925,0|HGLibA_52991,0</td></tr>
<tr><td>KLF8</td><td>4485</td><td>3</td><td>HGLibA_25083,1315|HGLibB_25049,1933|HGLibB_25050,1237|HGLibA_25085,0|HGLibA_25084,0|HGLibB_25048,0</td></tr>
<tr><td>ZMYND19</td><td>4417</td><td>3</td><td>HGLibA_55424,28|HGLibB_55356,736|HGLibB_55357,3653|HGLibA_55423,0|HGLibB_55355,0|HGLibA_55422,0</td></tr>
<tr><td>ME1</td><td>4403</td><td>3</td><td>HGLibA_28708,779|HGLibB_28668,1364|HGLibB_28667,2260|HGLibA_28709,0|HGLibA_28710,0|HGLibB_28669,0</td></tr>
<tr><td>FAM65C</td><td>4336</td><td>3</td><td>HGLibB_16651,1403|HGLibA_16674,1493|HGLibB_16650,1440|HGLibB_16652,0|HGLibA_16675,0|HGLibA_16673,0</td></tr>
<tr><td>NEU4</td><td>4241</td><td>3</td><td>HGLibA_31718,1331|HGLibA_31720,1399|HGLibB_31675,1511|HGLibB_31677,0|HGLibA_31719,0|HGLibB_31676,0</td></tr>
<tr><td>DIRAS1</td><td>4241</td><td>3</td><td>HGLibA_13255,13|HGLibB_13238,2835|HGLibB_13239,1393|HGLibA_13256,0|HGLibA_13257,0|HGLibB_13237,0</td></tr>
<tr><td>hsa-mir-496</td><td>4187</td><td>3</td><td>HGLibA_61384,1339|HGLibA_61385,1685|HGLibA_61383,1163|HGLibA_61386,0</td></tr>
<tr><td>AFG3L2</td><td>4179</td><td>3</td><td>HGLibB_01115,80|HGLibA_01117,2704|HGLibB_01117,1395|HGLibA_01118,0|HGLibA_01119,0|HGLibB_01116,0</td></tr>
<tr><td>hsa-mir-7704</td><td>4175</td><td>3</td><td>HGLibA_63951,1318|HGLibA_63950,1438|HGLibA_63952,1419|HGLibA_63953,0</td></tr>
<tr><td>RNF151</td><td>4151</td><td>3</td><td>HGLibB_41494,2675|HGLibB_41495,1473|HGLibA_41547,3|HGLibA_41548,0|HGLibA_41546,0|HGLibB_41496,0</td></tr>
<tr><td>PARD6B</td><td>4150</td><td>3</td><td>HGLibB_35142,1152|HGLibA_35186,1713|HGLibA_35188,1285|HGLibB_35140,0|HGLibA_35187,0|HGLibB_35141,0</td></tr>
<tr><td>GSTT2B</td><td>4144</td><td>3</td><td>HGLibB_20474,1444|HGLibA_20502,1389|HGLibB_20475,1311|HGLibA_20503,0|HGLibA_20501,0</td></tr>
<tr><td>B9D1</td><td>4064</td><td>3</td><td>HGLibB_04042,1291|HGLibB_04041,1427|HGLibA_04044,1346|HGLibB_04043,0|HGLibA_04045,0|HGLibA_04043,0</td></tr>
<tr><td>ENDOU</td><td>4047</td><td>3</td><td>HGLibA_15118,1394|HGLibB_15098,1285|HGLibA_15120,1368|HGLibB_15096,0|HGLibA_15119,0|HGLibB_15097,0</td></tr>
<tr><td>SF3B5</td><td>4039</td><td>3</td><td>HGLibB_43663,2612|HGLibA_43715,715|HGLibA_43716,712|HGLibB_43662,0|HGLibA_43714,0|HGLibB_43661,0</td></tr>
<tr><td>CSTB</td><td>4016</td><td>3</td><td>HGLibB_11467,1236|HGLibA_11480,5|HGLibB_11468,2775|HGLibA_11479,0|HGLibA_11481,0|HGLibB_11469,0</td></tr>
<tr><td>HIGD1B</td><td>3999</td><td>3</td><td>HGLibA_21310,2518|HGLibB_21280,171|HGLibB_21282,1310|HGLibB_21281,0|HGLibA_21309,0|HGLibA_21308,0</td></tr>
<tr><td>SLC44A2</td><td>3995</td><td>3</td><td>HGLibB_45108,1373|HGLibA_45159,1385|HGLibB_45106,1237|HGLibA_45160,0|HGLibB_45107,0|HGLibA_45161,0</td></tr>
<tr><td>TPK1</td><td>3949</td><td>3</td><td>HGLibB_51074,1236|HGLibA_51137,1291|HGLibA_51139,1422|HGLibA_51138,0|HGLibB_51075,0|HGLibB_51076,0</td></tr>
<tr><td>MPV17</td><td>3947</td><td>3</td><td>HGLibB_29742,1361|HGLibB_29741,2480|HGLibA_29784,106|HGLibA_29782,0|HGLibB_29743,0|HGLibA_29783,0</td></tr>
<tr><td>C10orf67</td><td>3941</td><td>3</td><td>HGLibA_05085,2|HGLibB_05081,269|HGLibB_05080,3670|HGLibA_05086,0|HGLibB_05082,0|HGLibA_05084,0</td></tr>
<tr><td>ANKEF1</td><td>3935</td><td>3</td><td>HGLibA_01953,1248|HGLibB_01949,1410|HGLibB_01951,1277|HGLibA_01951,0|HGLibB_01950,0|HGLibA_01952,0</td></tr>
<tr><td>DMAP1</td><td>3931</td><td>3</td><td>HGLibB_13371,3517|HGLibA_13389,212|HGLibB_13372,202|HGLibA_13391,0|HGLibA_13390,0|HGLibB_13370,0</td></tr>
<tr><td>TRO</td><td>3929</td><td>3</td><td>HGLibB_51652,2580|HGLibB_51651,1322|HGLibA_51717,27|HGLibA_51716,0|HGLibA_51715,0|HGLibB_51650,0</td></tr>
<tr><td>BAI3</td><td>3913</td><td>3</td><td>HGLibA_04120,1402|HGLibB_04116,1201|HGLibB_04117,1310|HGLibB_04118,0|HGLibA_04118,0|HGLibA_04119,0</td></tr>
<tr><td>CD8A</td><td>3886</td><td>3</td><td>HGLibA_08471,26|HGLibB_08464,2571|HGLibB_08465,1289|HGLibA_08472,0|HGLibA_08470,0|HGLibB_08463,0</td></tr>
<tr><td>RBMY1D</td><td>3877</td><td>3</td><td>HGLibB_40567,1345|HGLibA_40616,1307|HGLibB_40566,1225|HGLibA_40617,0</td></tr>
<tr><td>DHRSX_Y</td><td>3834</td><td>3</td><td>HGLibB_13141,1939|HGLibA_13160,523|HGLibA_13161,1372|HGLibB_13142,0|HGLibB_13143,0</td></tr>
<tr><td>TMEM139</td><td>3826</td><td>3</td><td>HGLibB_49897,193|HGLibB_49898,1345|HGLibA_49958,2288|HGLibB_49899,0|HGLibA_49959,0|HGLibA_49957,0</td></tr>
<tr><td>KANK4</td><td>3799</td><td>3</td><td>HGLibB_23998,330|HGLibA_24033,1228|HGLibB_24000,2241|HGLibA_24032,0|HGLibB_23999,0|HGLibA_24034,0</td></tr>
<tr><td>OSBPL7</td><td>3799</td><td>3</td><td>HGLibA_34665,1194|HGLibB_34620,1366|HGLibB_34618,1239|HGLibA_34663,0|HGLibA_34664,0|HGLibB_34619,0</td></tr>
<tr><td>C3orf55</td><td>3752</td><td>3</td><td>HGLibB_06247,2|HGLibB_06248,2537|HGLibB_06246,1213|HGLibA_06252,0|HGLibA_06251,0|HGLibA_06250,0</td></tr>
<tr><td>RCL1</td><td>3721</td><td>3</td><td>HGLibB_40646,1584|HGLibA_40698,1500|HGLibA_40697,637|HGLibA_40699,0|HGLibB_40648,0|HGLibB_40647,0</td></tr>
<tr><td>WIBG</td><td>3717</td><td>3</td><td>HGLibB_54232,2|HGLibA_54297,179|HGLibB_54231,3536|HGLibA_54298,0|HGLibB_54233,0|HGLibA_54299,0</td></tr>
<tr><td>OR4F17</td><td>3688</td><td>3</td><td>HGLibA_33995,14|HGLibA_33996,2666|HGLibA_33994,1008|HGLibB_33949,0|HGLibB_33951,0</td></tr>
<tr><td>FCRL5</td><td>3680</td><td>3</td><td>HGLibA_17238,1379|HGLibB_17213,1214|HGLibB_17215,1087|HGLibB_17214,0|HGLibA_17236,0|HGLibA_17237,0</td></tr>
<tr><td>FUT6</td><td>3660</td><td>3</td><td>HGLibB_18174,1331|HGLibB_18176,1140|HGLibB_18175,1189|HGLibA_18199,0|HGLibA_18201,0|HGLibA_18200,0</td></tr>
<tr><td>PHYHIPL</td><td>3644</td><td>3</td><td>HGLibB_36430,1005|HGLibA_36478,1562|HGLibB_36429,1077|HGLibA_36477,0|HGLibB_36431,0|HGLibA_36476,0</td></tr>
<tr><td>TMEM156</td><td>3629</td><td>3</td><td>HGLibB_49951,1191|HGLibB_49952,577|HGLibB_49953,1861|HGLibA_50013,0|HGLibA_50011,0|HGLibA_50012,0</td></tr>
<tr><td>HRCT1</td><td>3619</td><td>3</td><td>HGLibA_22088,194|HGLibA_22087,2375|HGLibA_22089,1050|HGLibB_22058,0|HGLibB_22059,0|HGLibB_22060,0</td></tr>
<tr><td>SLC10A5</td><td>3576</td><td>3</td><td>HGLibA_44341,1|HGLibB_44290,1053|HGLibB_44289,2522|HGLibA_44342,0|HGLibA_44343,0|HGLibB_44288,0</td></tr>
<tr><td>MRI1</td><td>3559</td><td>3</td><td>HGLibA_29858,53|HGLibA_29857,2430|HGLibB_29818,1076|HGLibA_29859,0|HGLibB_29816,0|HGLibB_29817,0</td></tr>
<tr><td>C2orf71</td><td>3543</td><td>3</td><td>HGLibB_06170,919|HGLibA_06172,1327|HGLibB_06168,1297|HGLibB_06169,0|HGLibA_06173,0|HGLibA_06174,0</td></tr>
<tr><td>hsa-mir-1273</td><td>3514</td><td>3</td><td>HGLibA_57535,4|HGLibA_57536,611|HGLibA_57534,2899|HGLibA_57537,0</td></tr>
</table>

<table>
<tr><td>GRIK3</td><td>3507</td><td>3</td><td>HGLibA_20286,1|HGLibA_20287,2109|HGLibA_20288,1397|HGLibB_20258,0|HGLibB_20260,0|HGLibB_20259,0</td></tr>
<tr><td>GPR37L1</td><td>3474</td><td>3</td><td>HGLibA_20035,1431|HGLibA_20036,689|HGLibB_20006,1354|HGLibB_20008,0|HGLibA_20034,0|HGLibB_20007,0</td></tr>
<tr><td>EGFR</td><td>3473</td><td>3</td><td>HGLibB_14613,1375|HGLibB_14615,1359|HGLibA_14636,739|HGLibB_14614,0|HGLibA_14635,0|HGLibA_14637,0</td></tr>
<tr><td>CACNB2</td><td>3463</td><td>3</td><td>HGLibA_06920,6|HGLibA_06919,3316|HGLibB_06915,141|HGLibB_06916,0|HGLibA_06921,0|HGLibB_06914,0</td></tr>
<tr><td>FAM213A</td><td>3446</td><td>3</td><td>HGLibB_16450,1126|HGLibA_16472,332|HGLibB_16448,1988|HGLibB_16449,0|HGLibA_16471,0|HGLibA_16470,0</td></tr>
<tr><td>OTUB2</td><td>3445</td><td>3</td><td>HGLibB_34710,19|HGLibB_34709,1031|HGLibA_34757,2395|HGLibB_34711,0|HGLibA_34756,0|HGLibA_34755,0</td></tr>
<tr><td>DLG1</td><td>3396</td><td>3</td><td>HGLibA_13324,675|HGLibB_13306,1683|HGLibA_13325,1038|HGLibB_13305,0|HGLibA_13323,0|HGLibB_13307,0</td></tr>
<tr><td>FAM107B</td><td>3384</td><td>3</td><td>HGLibB_15980,1019|HGLibA_16004,1164|HGLibB_15981,1201|HGLibA_16003,0|HGLibA_16002,0|HGLibB_15982,0</td></tr>
<tr><td>KCNN4</td><td>3381</td><td>3</td><td>HGLibA_24363,23|HGLibB_24328,1534|HGLibB_24329,1824|HGLibA_24364,0|HGLibB_24330,0|HGLibA_24362,0</td></tr>
<tr><td>SNX18</td><td>3378</td><td>3</td><td>HGLibB_45994,33|HGLibA_46051,1939|HGLibB_45996,1406|HGLibB_45995,0|HGLibA_46052,0|HGLibA_46050,0</td></tr>
<tr><td>FBXL22</td><td>3376</td><td>3</td><td>HGLibB_16981,1325|HGLibB_16979,640|HGLibB_16980,1411|HGLibA_17003,0|HGLibA_17002,0|HGLibA_17004,0</td></tr>
<tr><td>RAX</td><td>3351</td><td>3</td><td>HGLibB_40355,1357|HGLibA_40408,898|HGLibB_40356,1096|HGLibA_40406,0|HGLibA_40407,0|HGLibB_40357,0</td></tr>
<tr><td>DMGDH</td><td>3318</td><td>3</td><td>HGLibB_13385,1302|HGLibB_13386,1379|HGLibB_13387,637|HGLibA_13406,0|HGLibA_13404,0|HGLibA_13405,0</td></tr>
<tr><td>GCNT1</td><td>3246</td><td>3</td><td>HGLibB_18783,1027|HGLibA_18808,917|HGLibA_18809,1302|HGLibB_18785,0|HGLibA_18810,0|HGLibB_18784,0</td></tr>
<tr><td>TBPL1</td><td>3222</td><td>3</td><td>HGLibB_48566,1376|HGLibB_48568,863|HGLibA_48626,983|HGLibA_48625,0|HGLibA_48627,0|HGLibB_48567,0</td></tr>
<tr><td>ID3</td><td>3198</td><td>3</td><td>HGLibB_22500,1788|HGLibB_22498,6|HGLibA_22529,1404|HGLibB_22499,0|HGLibA_22531,0|HGLibA_22530,0</td></tr>
<tr><td>VPS54</td><td>3184</td><td>3</td><td>HGLibB_53745,1459|HGLibB_53747,544|HGLibA_53813,1181|HGLibB_53746,0|HGLibA_53812,0|HGLibA_53811,0</td></tr>
<tr><td>LRRC72</td><td>3181</td><td>3</td><td>HGLibA_27422,1340|HGLibB_27383,668|HGLibB_27381,1173|HGLibA_27421,0|HGLibB_27382,0|HGLibA_27423,0</td></tr>
<tr><td>CEACAM20</td><td>3176</td><td>3</td><td>HGLibA_08932,1216|HGLibB_08924,1307|HGLibB_08925,653|HGLibA_08933,0|HGLibB_08926,0|HGLibA_08931,0</td></tr>
<tr><td>CALB1</td><td>3171</td><td>3</td><td>HGLibB_06982,1303|HGLibA_06985,635|HGLibB_06981,1233|HGLibA_06987,0|HGLibB_06980,0|HGLibA_06986,0</td></tr>
<tr><td>APRT</td><td>3170</td><td>3</td><td>HGLibB_02570,1389|HGLibA_02571,1480|HGLibB_02571,301|HGLibA_02573,0|HGLibB_02569,0|HGLibA_02572,0</td></tr>
<tr><td>ADCK2</td><td>3145</td><td>3</td><td>HGLibB_00891,875|HGLibA_00894,1128|HGLibA_00893,1142|HGLibB_00892,0|HGLibA_00892,0|HGLibB_00890,0</td></tr>
<tr><td>APLP1</td><td>3144</td><td>3</td><td>HGLibB_02438,359|HGLibA_02440,2485|HGLibA_02439,300|HGLibB_02439,0|HGLibB_02437,0|HGLibA_02441,0</td></tr>
<tr><td>DEFB105A</td><td>3110</td><td>3</td><td>HGLibB_12810,1|HGLibB_12811,1243|HGLibB_12812,1866</td></tr>
<tr><td>HEBP2</td><td>3109</td><td>3</td><td>HGLibA_21087,2|HGLibB_21058,1076|HGLibB_21060,2031|HGLibA_21086,0|HGLibB_21059,0|HGLibA_21088,0</td></tr>
<tr><td>DIRC1</td><td>3099</td><td>3</td><td>HGLibB_13248,1366|HGLibB_13247,1122|HGLibB_13246,611|HGLibA_13265,0|HGLibA_13264,0|HGLibA_13266,0</td></tr>
<tr><td>AKR1B1</td><td>3064</td><td>3</td><td>HGLibA_01472,1242|HGLibB_01469,1148|HGLibB_01470,674|HGLibA_01473,0|HGLibB_01471,0|HGLibA_01471,0</td></tr>
<tr><td>USP39</td><td>3049</td><td>3</td><td>HGLibA_53356,1459|HGLibB_53289,1319|HGLibA_53357,271|HGLibB_53291,0|HGLibA_53355,0|HGLibB_53290,0</td></tr>
<tr><td>MID1IP1</td><td>3043</td><td>3</td><td>HGLibB_29192,1343|HGLibA_29234,308|HGLibB_29194,1392|HGLibA_29235,0|HGLibA_29233,0|HGLibB_29193,0</td></tr>
<tr><td>CFHR1</td><td>3040</td><td>3</td><td>HGLibB_09254,1319|HGLibB_09256,1048|HGLibA_09264,673|HGLibA_09263,0|HGLibA_09262,0|HGLibB_09255,0</td></tr>
<tr><td>CAPN5</td><td>3017</td><td>3</td><td>HGLibB_07163,312|HGLibB_07164,1768|HGLibB_07165,937|HGLibA_07168,0|HGLibA_07169,0|HGLibA_07170,0</td></tr>
<tr><td>CDCP1</td><td>2998</td><td>3</td><td>HGLibA_08623,2621|HGLibB_08617,281|HGLibA_08624,96|HGLibB_08615,0|HGLibA_08622,0|HGLibB_08616,0</td></tr>
<tr><td>NLRP2</td><td>2996</td><td>3</td><td>HGLibA_32115,1767|HGLibB_32072,1|HGLibB_32071,1228|HGLibA_32114,0|HGLibB_32073,0|HGLibA_32116,0</td></tr>
<tr><td>C15orf26</td><td>2985</td><td>3</td><td>HGLibA_05378,620|HGLibA_05379,1248|HGLibA_05377,1117|HGLibB_05375,0|HGLibB_05374,0|HGLibB_05373,0</td></tr>
<tr><td>FDFT1</td><td>2960</td><td>3</td><td>HGLibA_17252,48|HGLibB_17228,2181|HGLibA_17251,731|HGLibA_17253,0|HGLibB_17230,0|HGLibB_17229,0</td></tr>
<tr><td>MICB</td><td>2953</td><td>3</td><td>HGLibB_29179,1383|HGLibA_29220,1329|HGLibB_29178,241|HGLibA_29219,0|HGLibA_29218,0|HGLibB_29177,0</td></tr>
<tr><td>ABHD17C</td><td>2951</td><td>3</td><td>HGLibB_00268,1396|HGLibB_00269,328|HGLibA_00268,1227|HGLibA_00270,0|HGLibB_00270,0|HGLibA_00269,0</td></tr>
<tr><td>PSG11</td><td>2950</td><td>3</td><td>HGLibB_38988,1804|HGLibB_38987,973|HGLibA_39036,173|HGLibA_39038,0|HGLibB_38989,0|HGLibA_39037,0</td></tr>
<tr><td>SCARB2</td><td>2947</td><td>3</td><td>HGLibB_42818,956|HGLibA_42873,1952|HGLibB_42819,39|HGLibA_42872,0|HGLibB_42820,0|HGLibA_42871,0</td></tr>
<tr><td>OR2H1</td><td>2947</td><td>3</td><td>HGLibB_33758,1167|HGLibA_33803,812|HGLibA_33802,968|HGLibA_33804,0|HGLibB_33759,0|HGLibB_33757,0</td></tr>
<tr><td>TTLL7</td><td>2930</td><td>3</td><td>HGLibB_52147,1274|HGLibA_52213,1135|HGLibA_52212,521|HGLibB_52148,0|HGLibB_52149,0|HGLibA_52214,0</td></tr>
<tr><td>MYOM2</td><td>2921</td><td>3</td><td>HGLibB_30849,1494|HGLibB_30851,1426|HGLibB_30850,1|HGLibA_30891,0|HGLibA_30893,0|HGLibA_30892,0</td></tr>
<tr><td>ZNF71</td><td>2920</td><td>3</td><td>HGLibA_56609,2547|HGLibB_56542,232|HGLibB_56540,141|HGLibB_56541,0|HGLibA_56607,0|HGLibA_56608,0</td></tr>
<tr><td>PMAIP1</td><td>2909</td><td>3</td><td>HGLibA_37292,1098|HGLibA_37290,1180|HGLibB_37243,631|HGLibB_37242,0|HGLibA_37291,0|HGLibB_37241,0</td></tr>
<tr><td>SCRIB</td><td>2890</td><td>3</td><td>HGLibB_42993,1274|HGLibA_43047,933|HGLibB_42994,683|HGLibA_43045,0|HGLibB_42992,0|HGLibA_43046,0</td></tr>
<tr><td>CCL8</td><td>2889</td><td>3</td><td>HGLibA_08042,580|HGLibB_08035,1232|HGLibB_08034,1077|HGLibB_08036,0|HGLibA_08041,0|HGLibA_08043,0</td></tr>
<tr><td>NDUFS5</td><td>2886</td><td>3</td><td>HGLibB_31516,1147|HGLibA_31556,1401|HGLibA_31558,338|HGLibB_31514,0|HGLibA_31557,0|HGLibB_31515,0</td></tr>
<tr><td>PARP8</td><td>2881</td><td>3</td><td>HGLibB_35196,1445|HGLibA_35244,1401|HGLibA_35245,35|HGLibB_35197,0|HGLibB_35198,0|HGLibA_35243,0</td></tr>
<tr><td>AAR2</td><td>2861</td><td>3</td><td>HGLibA_00062,2047|HGLibA_00061,114|HGLibB_00062,700|HGLibB_00061,0|HGLibA_00063,0|HGLibB_00063,0</td></tr>
<tr><td>ARID3C</td><td>2856</td><td>3</td><td>HGLibA_02898,27|HGLibA_02897,2808|HGLibA_02896,21|HGLibB_02896,0|HGLibB_02894,0|HGLibB_02895,0</td></tr>
<tr><td>PLAGL2</td><td>2851</td><td>3</td><td>HGLibB_36909,1645|HGLibB_36910,1033|HGLibA_36957,173|HGLibB_36911,0|HGLibA_36956,0|HGLibA_36958,0</td></tr>
<tr><td>ANKRD35</td><td>2840</td><td>3</td><td>HGLibA_02084,1477|HGLibB_02083,1361|HGLibB_02081,2|HGLibA_02083,0|HGLibA_02085,0|HGLibB_02082,0</td></tr>
<tr><td>PHF15</td><td>2839</td><td>3</td><td>HGLibB_36301,1397|HGLibA_36349,169|HGLibA_36348,1273|HGLibB_36302,0|HGLibA_36347,0|HGLibB_36300,0</td></tr>
<tr><td>IFT20</td><td>2835</td><td>3</td><td>HGLibB_22721,912|HGLibA_22752,31|HGLibB_22722,1892|HGLibB_22720,0|HGLibA_22753,0|HGLibA_22751,0</td></tr>
<tr><td>SHKBP1</td><td>2830</td><td>3</td><td>HGLibB_44004,1116|HGLibA_44056,1708|HGLibA_44058,6|HGLibA_44057,0|HGLibB_44005,0|HGLibB_44003,0</td></tr>
<tr><td>SATB2</td><td>2827</td><td>3</td><td>HGLibA_42780,2772|HGLibB_42727,10|HGLibA_42778,45|HGLibB_42725,0|HGLibA_42779,0|HGLibB_42726,0</td></tr>
<tr><td>OR8I2</td><td>2820</td><td>3</td><td>HGLibA_34535,300|HGLibA_34536,1218|HGLibB_34491,1302|HGLibA_34534,0|HGLibB_34489,0|HGLibB_34490,0</td></tr>
<tr><td>TMEM106C</td><td>2818</td><td>3</td><td>HGLibB_49798,2714|HGLibA_49859,29|HGLibB_49800,75|HGLibB_49799,0|HGLibA_49860,0|HGLibA_49858,0</td></tr>
<tr><td>SPA17</td><td>2813</td><td>3</td><td>HGLibB_46266,1487|HGLibA_46320,1291|HGLibB_46265,35|HGLibA_46321,0|HGLibA_46322,0|HGLibB_46264,0</td></tr>
<tr><td>ACOT13</td><td>2806</td><td>3</td><td>HGLibA_00476,327|HGLibB_00476,1266|HGLibB_00477,1213|HGLibA_00475,0|HGLibB_00475,0|HGLibA_00477,0</td></tr>
<tr><td>TMUB2</td><td>2803</td><td>3</td><td>HGLibB_50622,317|HGLibA_50686,2448|HGLibA_50684,38|HGLibA_50685,0|HGLibB_50624,0|HGLibB_50623,0</td></tr>
<tr><td>HIF1AN</td><td>2795</td><td>3</td><td>HGLibB_21271,1402|HGLibA_21301,1382|HGLibA_21299,11|HGLibB_21273,0|HGLibB_21272,0|HGLibA_21300,0</td></tr>
<tr><td>PKHD1</td><td>2794</td><td>3</td><td>HGLibB_36766,389|HGLibB_36765,2403|HGLibA_36814,2|HGLibA_36812,0|HGLibA_36813,0|HGLibB_36767,0</td></tr>
<tr><td>ENPP7</td><td>2788</td><td>3</td><td>HGLibB_15163,26|HGLibB_15164,1367|HGLibB_15162,1395|HGLibA_15185,0|HGLibA_15184,0|HGLibA_15186,0</td></tr>
<tr><td>ISCA1</td><td>2780</td><td>3</td><td>HGLibB_23614,1298|HGLibB_23612,326|HGLibA_23645,1156|HGLibA_23647,0|HGLibB_23613,0|HGLibA_23646,0</td></tr>
<tr><td>C1RL</td><td>2776</td><td>3</td><td>HGLibB_05968,19|HGLibB_05969,37|HGLibA_05972,2720|HGLibA_05971,0|HGLibA_05973,0|HGLibB_05967,0</td></tr>
<tr><td>TPO</td><td>2767</td><td>3</td><td>HGLibB_51094,90|HGLibB_51093,2676|HGLibA_51157,1|HGLibA_51155,0|HGLibA_51156,0|HGLibB_51092,0</td></tr>
<tr><td>MAD1L1</td><td>2766</td><td>3</td><td>HGLibA_27842,1419|HGLibA_27843,1187|HGLibB_27800,160|HGLibB_27801,0|HGLibB_27802,0|HGLibA_27841,0</td></tr>
<tr><td>NOD1</td><td>2764</td><td>3</td><td>HGLibB_32201,1349|HGLibB_32202,20|HGLibA_32245,1395|HGLibB_32200,0|HGLibA_32244,0|HGLibA_32243,0</td></tr>
<tr><td>SKIV2L2</td><td>2761</td><td>3</td><td>HGLibB_44234,1298|HGLibB_44235,150|HGLibA_44287,1313|HGLibA_44289,0|HGLibA_44288,0|HGLibB_44236,0</td></tr>
<tr><td>TRIOBP</td><td>2759</td><td>3</td><td>HGLibB_51565,1228|HGLibB_51567,195|HGLibA_51631,1336|HGLibA_51630,0|HGLibB_51566,0|HGLibA_51629,0</td></tr>
<tr><td>OR5M3</td><td>2758</td><td>3</td><td>HGLibB_34293,344|HGLibB_34292,1210|HGLibB_34291,1204|HGLibA_34336,0|HGLibA_34338,0|HGLibA_34337,0</td></tr>
<tr><td>SMURF1</td><td>2751</td><td>3</td><td>HGLibB_45789,86|HGLibA_45845,1382|HGLibB_45790,1283|HGLibA_45846,0|HGLibB_45791,0|HGLibA_45844,0</td></tr>
<tr><td>CATSPERB</td><td>2742</td><td>3</td><td>HGLibA_07368,1788|HGLibB_07360,941|HGLibA_07366,13|HGLibB_07362,0|HGLibB_07361,0|HGLibA_07367,0</td></tr>
<tr><td>CLASP1</td><td>2734</td><td>3</td><td>HGLibB_09783,363|HGLibB_09784,1178|HGLibB_09785,1193|HGLibA_09794,0|HGLibA_09793,0|HGLibA_09792,0</td></tr>
<tr><td>PRDX6</td><td>2732</td><td>3</td><td>HGLibA_38421,164|HGLibA_38422,20|HGLibB_38373,2548|HGLibB_38374,0|HGLibB_38372,0|HGLibA_38423,0</td></tr>
<tr><td>MSRB3</td><td>2724</td><td>3</td><td>HGLibB_30206,164|HGLibB_30207,1278|HGLibA_30247,1282|HGLibA_30246,0|HGLibB_30205,0|HGLibA_30248,0</td></tr>
</table>

PEX2	2707	3	HGLibA_36107,2392|HGLibA_36108,48|HGLibB_36060,267|HGLibB_36062,0|HGLibB_36061,0|HGLibA_36109,0
TMEM244	2697	3	HGLibA_50297,1361|HGLibB_50236,1297|HGLibB_50238,39|HGLibB_50237,0|HGLibA_50296,0|HGLibA_50298,0
S100A2	2693	3	HGLibA_42551,1184|HGLibB_42498,309|HGLibB_42497,1200|HGLibB_42499,0|HGLibA_42550,0|HGLibA_42552,0
TRIM60	2683	3	HGLibB_51500,6|HGLibA_51564,1315|HGLibB_51501,1362|HGLibA_51565,0|HGLibA_51563,0|HGLibB_51499,0
OR10Q1	2662	3	HGLibB_33495,11|HGLibB_33493,1317|HGLibB_33494,1334|HGLibA_33539,0|HGLibA_33538,0|HGLibA_33540,0
OR1A2	2656	3	HGLibB_33599,94|HGLibA_33644,1187|HGLibB_33598,1375|HGLibA_33643,0|HGLibA_33645,0|HGLibB_33600,0
MASP2	2648	3	HGLibA_28396,1335|HGLibA_28397,27|HGLibB_28355,1286|HGLibA_28398,0|HGLibB_28357,0|HGLibB_28356,0
ENTHD1	2646	3	HGLibB_15168,1308|HGLibA_15192,6|HGLibB_15169,1332|HGLibA_15191,0|HGLibB_15170,0|HGLibA_15190,0
CNRIP1	2643	3	HGLibA_10379,166|HGLibB_10369,253|HGLibB_10368,2224|HGLibA_10381,0|HGLibA_10380,0|HGLibB_10370,0
CHST8	2632	3	HGLibB_09640,1242|HGLibA_09650,35|HGLibB_09639,1355|HGLibA_09648,0|HGLibA_09649,0|HGLibB_09641,0
FNDC7	2628	3	HGLibB_17733,1357|HGLibA_17756,10|HGLibA_17755,1261|HGLibA_17757,0|HGLibB_17732,0|HGLibB_17734,0
WDFY1	2627	3	HGLibA_54003,217|HGLibB_53937,687|HGLibA_54004,1723|HGLibB_53938,0|HGLibA_54005,0|HGLibB_53939,0
STX3	2625	3	HGLibB_47589,2615|HGLibA_47646,1|HGLibB_47588,9|HGLibB_47587,0|HGLibA_47645,0|HGLibA_47644,0
HLA-DOB	2623	3	HGLibB_21583,1311|HGLibA_21612,1307|HGLibA_21611,5|HGLibB_21585,0|HGLibA_21613,0|HGLibB_21584,0
NR4A3	2622	3	HGLibB_32588,1153|HGLibB_32587,38|HGLibA_32631,1431|HGLibB_32586,0|HGLibA_32632,0|HGLibA_32630,0
QARS	2622	3	HGLibB_39668,1283|HGLibB_39667,704|HGLibA_39719,635|HGLibA_39718,0|HGLibB_39666,0|HGLibA_39717,0
MNDA	2621	3	HGLibB_29525,1843|HGLibA_29566,777|HGLibB_29526,1|HGLibA_29567,0|HGLibA_29568,0|HGLibB_29527,0
NLRP9	2621	3	HGLibA_32135,1282|HGLibA_32137,132|HGLibB_32093,1207|HGLibB_32092,0|HGLibB_32094,0|HGLibA_32136,0
IL16	2616	3	HGLibB_22954,1240|HGLibB_22956,3|HGLibB_22955,1373|HGLibA_22986,0|HGLibA_22985,0|HGLibA_22987,0
hsa-mir-807:	2607	3	HGLibA_64133,1238|HGLibA_64135,1367|HGLibA_64136,2|HGLibA_64134,0
SFTA2	2598	3	HGLibB_43706,1375|HGLibB_43708,1221|HGLibB_43707,2|HGLibA_43759,0|HGLibA_43760,0|HGLibA_43761,0
SLC37A2	2589	3	HGLibA_45045,2529|HGLibA_45046,24|HGLibA_45047,36|HGLibB_44993,0|HGLibB_44992,0|HGLibB_44994,0
NME3	2580	3	HGLibA_32161,1363|HGLibB_32116,1193|HGLibB_32118,24|HGLibA_32159,0|HGLibA_32160,0|HGLibB_32117,0
AP3B1	2573	3	HGLibB_02317,1255|HGLibA_02319,763|HGLibB_02319,555|HGLibA_02321,0|HGLibA_02320,0|HGLibB_02318,0
ZNF334	2567	3	HGLibA_55823,1930|HGLibB_55754,145|HGLibB_55755,492|HGLibB_55756,0|HGLibA_55821,0|HGLibA_55822,0
SEMA3B	2547	3	HGLibA_43321,1|HGLibB_43268,43|HGLibB_43270,2503|HGLibB_43269,0|HGLibA_43323,0|HGLibA_43322,0
VWDE	2546	3	HGLibB_53867,653|HGLibA_53932,1247|HGLibB_53865,646|HGLibB_53866,0|HGLibA_53933,0|HGLibA_53931,0
NCOA2	2545	3	HGLibB_31296,1311|HGLibA_31339,1184|HGLibB_31295,50|HGLibB_31297,0|HGLibA_31337,0|HGLibA_31338,0
EXD2	2544	3	HGLibB_15696,1307|HGLibB_15698,13|HGLibB_15697,1224|HGLibA_15720,0|HGLibA_15718,0|HGLibA_15719,0
ZMAT2	2544	3	HGLibB_55301,1071|HGLibB_55303,94|HGLibA_55369,1379|HGLibA_55370,0|HGLibA_55368,0|HGLibB_55302,0
GDAP1	2544	3	HGLibA_18838,1283|HGLibA_18840,1218|HGLibB_18815,43|HGLibB_18813,0|HGLibA_18839,0|HGLibB_18814,0
PA2G4	2542	3	HGLibA_34914,75|HGLibA_34915,1098|HGLibB_34869,1369|HGLibA_34916,0|HGLibB_34870,0|HGLibB_34868,0
ACTR3	2534	3	HGLibB_00657,1151|HGLibA_00659,1350|HGLibB_00659,33|HGLibA_00660,0|HGLibB_00658,0|HGLibA_00658,0
NDUFA4	2529	3	HGLibA_31458,20|HGLibA_31459,1151|HGLibB_31416,1358|HGLibA_31457,0|HGLibB_31417,0|HGLibB_31415,0
IFT140	2518	3	HGLibB_22714,1518|HGLibA_22745,984|HGLibB_22716,16|HGLibB_22715,0|HGLibA_22746,0|HGLibA_22747,0
GCLM	2518	3	HGLibA_18798,173|HGLibB_18772,342|HGLibB_18771,2003|HGLibB_18773,0|HGLibA_18797,0|HGLibA_18796,0
ASB13	2515	3	HGLibA_03216,2|HGLibB_03212,1329|HGLibB_03213,1184|HGLibB_03214,0|HGLibA_03214,0|HGLibA_03215,0
PTPN1	2514	3	HGLibB_39381,1259|HGLibA_39431,1175|HGLibB_39382,80|HGLibB_39380,0|HGLibA_39429,0|HGLibA_39430,0
PTPRC	2512	3	HGLibA_39490,102|HGLibB_39440,1171|HGLibA_39489,1239|HGLibB_39441,0|HGLibB_39439,0|HGLibA_39491,0
PIGB	2504	3	HGLibB_36498,1475|HGLibA_36546,364|HGLibB_36499,665|HGLibA_36545,0|HGLibB_36500,0|HGLibA_36547,0
TIMELESS	2488	3	HGLibA_49490,1075|HGLibB_49429,1336|HGLibB_49431,77|HGLibA_49489,0|HGLibB_49430,0|HGLibA_49491,0
LPL	2484	3	HGLibA_27103,17|HGLibB_27065,1171|HGLibA_27104,1296|HGLibA_27105,0|HGLibB_27064,0|HGLibB_27063,0
KRT26	2477	3	HGLibA_25463,1166|HGLibB_25427,12|HGLibA_25461,1299|HGLibB_25425,0|HGLibA_25462,0|HGLibB_25426,0
BBS12	2473	3	HGLibB_04211,1274|HGLibB_04209,3|HGLibA_04213,1196|HGLibB_04210,0|HGLibA_04211,0|HGLibA_04212,0
ODF3	2470	3	HGLibB_33240,1221|HGLibB_33238,2|HGLibA_33283,1247|HGLibA_33284,0|HGLibB_33239,0|HGLibA_33285,0
C19orf21	2457	3	HGLibB_05633,3|HGLibB_05632,111|HGLibB_05631,2343
DNAH10	2449	3	HGLibA_13470,1392|HGLibB_13452,1040|HGLibB_13453,17|HGLibA_13471,0|HGLibB_13451,0|HGLibA_13472,0
TMBIM6	2448	3	HGLibB_49684,1270|HGLibB_49685,1157|HGLibA_49745,21|HGLibA_49746,0|HGLibA_49744,0|HGLibB_49686,0
MPZL3	2445	3	HGLibB_29761,1075|HGLibA_29800,1348|HGLibB_29760,22|HGLibA_29801,0|HGLibA_29802,0|HGLibB_29759,0
SERGEF	2445	3	HGLibA_43479,1134|HGLibB_43424,155|HGLibB_43426,1156|HGLibA_43477,0|HGLibB_43425,0|HGLibA_43478,0
TLE6	2444	3	HGLibB_49558,1199|HGLibA_49620,1235|HGLibB_49560,10|HGLibB_49559,0|HGLibA_49619,0|HGLibA_49618,0
PSEN1	2441	3	HGLibB_38975,49|HGLibB_38977,1331|HGLibA_39024,1061|HGLibA_39025,0|HGLibB_38976,0|HGLibA_39026,0
LOC339862	2435	3	HGLibB_26883,1235|HGLibB_26881,1181|HGLibA_26922,19|HGLibB_26882,0|HGLibA_26921,0|HGLibA_26920,0
SULT2A1	2430	3	HGLibB_47702,1130|HGLibB_47700,1297|HGLibB_47701,3|HGLibA_47759,0|HGLibA_47758,0|HGLibA_47757,0
C17orf99	2429	3	HGLibB_05600,7|HGLibB_05599,1330|HGLibA_05603,1092|HGLibA_05604,0|HGLibB_05598,0|HGLibA_05602,0
ABCC8	2427	3	HGLibB_00186,1076|HGLibB_00185,2|HGLibB_00184,1349|HGLibA_00185,0|HGLibA_00186,0|HGLibA_00184,0
PQLC3	2403	3	HGLibA_38257,663|HGLibB_38208,1100|HGLibB_38209,640|HGLibA_38256,0|HGLibA_38258,0|HGLibB_38207,0
HMSD	2381	3	HGLibB_21727,2045|HGLibB_21726,17|HGLibA_21757,319|HGLibA_21755,0|HGLibA_21756,0|HGLibB_21728,0
FAM171B	2380	3	HGLibA_16271,656|HGLibB_16247,78|HGLibB_16249,1646|HGLibA_16270,0|HGLibB_16248,0|HGLibA_16269,0
FLI1	2379	3	HGLibB_17592,1056|HGLibA_17616,316|HGLibA_17614,1007|HGLibB_17593,0|HGLibB_17591,0|HGLibA_17615,0
TSEN15	2378	3	HGLibA_51830,1110|HGLibA_51828,1238|HGLibB_51764,30|HGLibB_51765,0|HGLibB_51763,0|HGLibA_51829,0
POPDC3	2364	3	HGLibA_37702,86|HGLibA_37701,547|HGLibB_37653,1731|HGLibA_37703,0|HGLibB_37654,0|HGLibB_37652,0
SEMA3G	2361	3	HGLibB_43284,44|HGLibA_43336,2006|HGLibB_43285,311|HGLibA_43337,0|HGLibB_43283,0|HGLibA_43338,0
MRPS21	2346	3	HGLibB_30022,633|HGLibA_30061,191|HGLibB_30021,1522|HGLibB_30020,0|HGLibA_30063,0|HGLibA_30062,0
CTSS	2338	3	HGLibB_11717,40|HGLibB_11716,1138|HGLibA_11729,1160|HGLibB_11715,0|HGLibA_11728,0|HGLibA_11727,0
PTP4A1	2317	3	HGLibB_39354,851|HGLibA_39403,1435|HGLibB_39353,31|HGLibA_39402,0|HGLibA_39404,0|HGLibB_39355,0
PSMB6	2304	3	HGLibB_39067,1183|HGLibA_39115,2|HGLibB_39065,1119|HGLibA_39116,0|HGLibA_39114,0|HGLibB_39066,0
SLC26A3	2302	3	HGLibA_44791,1102|HGLibA_44793,1|HGLibB_44739,1199|HGLibA_44792,0|HGLibB_44738,0|HGLibB_44740,0
USP17L2	2289	3	HGLibB_53176,909|HGLibA_53241,2|HGLibB_53175,1378|HGLibA_53243,0|HGLibA_53242,0|HGLibB_53177,0
STMN4	2283	3	HGLibB_47468,185|HGLibB_47469,1685|HGLibB_47467,413|HGLibA_47525,0|HGLibA_47526,0|HGLibA_47524,0
USP47	2278	3	HGLibB_53314,1116|HGLibB_53313,35|HGLibA_53379,1127|HGLibA_53380,0|HGLibB_53315,0|HGLibA_53381,0
ZNF571	2274	3	HGLibB_56211,636|HGLibB_56212,293|HGLibB_56210,1345|HGLibA_56277,0|HGLibA_56279,0|HGLibA_56278,0
TMUB1	2254	3	HGLibA_50681,677|HGLibA_50683,1277|HGLibB_50619,300|HGLibB_50621,0|HGLibA_50682,0|HGLibB_50620,0
AMDHD2	2251	3	HGLibB_01762,2154|HGLibB_01761,96|HGLibA_01762,1|HGLibB_01760,0|HGLibA_01764,0|HGLibA_01763,0
LRIF1	2249	3	HGLibB_27133,603|HGLibB_27132,983|HGLibA_27173,663|HGLibA_27174,0|HGLibB_27134,0|HGLibA_27172,0
PDLIM1	2234	3	HGLibB_35861,307|HGLibA_35906,1263|HGLibA_35908,664|HGLibB_35860,0|HGLibA_35907,0|HGLibB_35859,0
PPIA	2230	3	HGLibA_37893,61|HGLibB_37845,1384|HGLibB_37846,785|HGLibB_37844,0|HGLibA_37895,0|HGLibA_37894,0

IKZF4	2229	3	HGLibB_22906,1047|HGLibA_22937,1180|HGLibB_22907,2|HGLibB_22908,0|HGLibA_22938,0|HGLibA_22939,0
SMAP2	2219	3	HGLibA_45600,1082|HGLibA_45599,1133|HGLibA_45598,4|HGLibB_45545,0|HGLibB_45543,0|HGLibB_45544,0
RAP2A	2213	3	HGLibA_40236,770|HGLibB_40185,1440|HGLibA_40237,3|HGLibB_40184,0|HGLibA_40235,0|HGLibB_40186,0
PTPRD	2211	3	HGLibA_39496,746|HGLibA_39497,84|HGLibB_39445,1381|HGLibB_39446,0|HGLibB_39447,0|HGLibA_39495,0
IQCD	2192	3	HGLibA_23495,605|HGLibB_23463,432|HGLibB_23462,1155|HGLibB_23464,0|HGLibA_23496,0|HGLibA_23497,0
PITPNM2	2183	3	HGLibA_36753,258|HGLibA_36754,1313|HGLibB_36705,612|HGLibB_36707,0|HGLibA_36752,0|HGLibB_36706,0
SNX24	2165	3	HGLibB_46013,244|HGLibA_46068,1330|HGLibB_46012,591|HGLibA_46069,0|HGLibA_46070,0|HGLibB_46014,0
GTF2H2D	2162	3	HGLibB_20525,498|HGLibB_20524,375|HGLibA_20552,1289|HGLibB_20526,0|HGLibA_20554,0
GPX4	2161	3	HGLibA_20179,120|HGLibA_20178,1725|HGLibB_20152,316|HGLibA_20180,0|HGLibB_20151,0|HGLibB_20150,0
EGF	2142	3	HGLibB_14598,1654|HGLibA_14622,483|HGLibA_14620,5|HGLibA_14621,0|HGLibB_14600,0|HGLibB_14599,0
GTPBP5	2135	3	HGLibA_20612,1290|HGLibB_20585,654|HGLibB_20584,191|HGLibA_20613,0|HGLibB_20586,0|HGLibA_20614,0
KCNJ14	2116	3	HGLibA_24260,660|HGLibA_24261,1438|HGLibB_24228,18|HGLibA_24262,0|HGLibB_24227,0|HGLibB_24226,0
TYK2	2115	3	HGLibB_52391,5|HGLibA_52457,2105|HGLibB_52392,5|HGLibA_52455,0|HGLibA_52456,0|HGLibB_52390,0
PHF5A	2104	3	HGLibB_36334,238|HGLibA_36381,1214|HGLibB_36335,652|HGLibA_36382,0|HGLibA_36380,0|HGLibB_36333,0
LOC649330	2102	3	HGLibB_26942,1694|HGLibA_26982,4|HGLibA_26980,404|HGLibB_26941,0|HGLibB_26940,0|HGLibA_26981,0
SULT1C3	2101	3	HGLibB_47692,612|HGLibA_47748,1278|HGLibB_47691,211|HGLibA_47749,0|HGLibB_47693,0|HGLibA_47750,0
C2CD4C	2101	3	HGLibA_06100,1524|HGLibB_06096,260|HGLibA_06102,317|HGLibA_06101,0|HGLibB_06098,0|HGLibB_06097,0
C7orf34	2099	3	HGLibB_06536,1228|HGLibA_06539,721|HGLibB_06535,150|HGLibB_06534,0|HGLibA_06540,0|HGLibA_06538,0
PDHB	2090	3	HGLibA_35868,614|HGLibA_35869,1309|HGLibA_35867,167|HGLibB_35820,0|HGLibB_35821,0|HGLibB_35822,0
CD180	2083	3	HGLibA_08258,662|HGLibA_08257,1415|HGLibA_08259,6|HGLibB_08251,0|HGLibB_08250,0|HGLibB_08252,0
CDS1	2073	3	HGLibA_08882,2|HGLibB_08875,704|HGLibB_08873,1367|HGLibA_08881,0|HGLibA_08880,0|HGLibB_08874,0
ANKAR	2059	3	HGLibB_01940,128|HGLibB_01941,1230|HGLibA_01944,701|HGLibB_01942,0|HGLibA_01943,0|HGLibA_01942,0
APOBEC3C	2056	3	HGLibA_02482,1391|HGLibB_02480,660|HGLibA_02483,5|HGLibB_02479,0|HGLibA_02481,0|HGLibB_02481,0
CCR3	2050	3	HGLibA_08152,562|HGLibA_08154,1485|HGLibB_08145,3|HGLibA_08153,0|HGLibB_08147,0|HGLibB_08146,0
AKAP6	2047	3	HGLibB_01437,552|HGLibA_01439,1315|HGLibA_01438,180|HGLibB_01436,0|HGLibA_01440,0|HGLibB_01438,0
PALM3	2047	3	HGLibA_35075,3|HGLibB_35029,1335|HGLibB_35030,709|HGLibA_35077,0|HGLibB_35031,0|HGLibA_35076,0
HTR4	2038	3	HGLibA_22394,43|HGLibB_22363,1289|HGLibA_22395,706|HGLibB_22364,0|HGLibA_22396,0|HGLibB_22365,0
VASP	2036	3	HGLibA_53538,613|HGLibB_53473,1408|HGLibB_53472,15|HGLibB_53474,0|HGLibA_53540,0|HGLibA_53539,0
RPUSD4	2032	3	HGLibA_42215,1400|HGLibB_42162,3|HGLibA_42216,629|HGLibB_42161,0|HGLibB_42163,0|HGLibA_42214,0
TMOD2	2028	3	HGLibB_50537,39|HGLibA_50598,652|HGLibB_50536,1337|HGLibA_50596,0|HGLibB_50538,0|HGLibA_50597,0
OR2T8	2023	3	HGLibB_33848,1447|HGLibA_33893,280|HGLibB_33847,296|HGLibB_33849,0|HGLibA_33892,0|HGLibA_33894,0
DYRK2	2019	3	HGLibB_14256,1219|HGLibB_14255,533|HGLibA_14276,267|HGLibA_14277,0|HGLibA_14278,0|HGLibB_14254,0
ANXA8	2016	3	HGLibA_02253,367|HGLibB_02250,1454|HGLibA_02251,195|HGLibB_02249,0|HGLibA_02252,0
TM9SF3	2003	3	HGLibB_49666,1166|HGLibB_49668,720|HGLibA_49728,117|HGLibB_49667,0|HGLibA_49726,0|HGLibA_49727,0
RALGDS	1997	3	HGLibB_40115,1406|HGLibB_40113,1|HGLibB_40114,590|HGLibA_40165,0|HGLibA_40164,0|HGLibA_40166,0
YY2	1982	3	HGLibA_54762,655|HGLibB_54697,614|HGLibB_54695,713|HGLibA_54763,0|HGLibB_54696,0|HGLibA_54764,0
AFF3	1979	3	HGLibB_01111,75|HGLibA_01112,1265|HGLibB_01109,639|HGLibB_01110,0|HGLibA_01113,0|HGLibA_01111,0
GALNT1	1979	3	HGLibB_18501,664|HGLibB_18502,1311|HGLibB_18503,4|HGLibA_18528,0|HGLibA_18527,0|HGLibA_18526,0
DNAJC16	1954	3	HGLibB_13562,565|HGLibB_13563,1179|HGLibA_13583,210|HGLibA_13582,0|HGLibA_13581,0|HGLibB_13564,0
FUCA1	1939	3	HGLibB_18130,1154|HGLibB_18129,693|HGLibA_18154,92|HGLibB_18131,0|HGLibA_18155,0|HGLibA_18156,0
KRT2	1939	3	HGLibB_25408,625|HGLibB_25409,1184|HGLibB_25407,130|HGLibA_25443,0|HGLibA_25444,0|HGLibA_25445,0
WDR89	1934	3	HGLibA_54211,1284|HGLibA_54210,644|HGLibB_54144,6|HGLibB_54145,0|HGLibB_54146,0|HGLibA_54212,0
PCDHB15	1929	3	HGLibB_35441,1059|HGLibB_35439,194|HGLibA_35487,676|HGLibB_35440,0|HGLibA_35488,0|HGLibA_35486,0
PRAP1	1925	3	HGLibB_38286,645|HGLibA_38335,21|HGLibB_38287,1259|HGLibB_38285,0|HGLibA_38336,0|HGLibA_38334,0
UNC5A	1913	3	HGLibB_52949,544|HGLibA_53013,1367|HGLibB_52948,2|HGLibB_52950,0|HGLibA_53015,0|HGLibA_53014,0
CASP10	1908	3	HGLibA_07297,1|HGLibB_07293,651|HGLibB_07291,1256|HGLibA_07299,0|HGLibB_07292,0|HGLibA_07298,0
TRAPPC1	1905	3	HGLibB_51235,52|HGLibA_51298,12|HGLibA_51296,1841|HGLibA_51297,0|HGLibB_51234,0|HGLibB_51233,0
STOML3	1904	3	HGLibB_47484,580|HGLibB_47483,21|HGLibA_47539,1303|HGLibA_47540,0|HGLibB_47482,0|HGLibA_47541,0
hsa-mir-642a	1901	3	HGLibA_62808,1233|HGLibA_62807,38|HGLibA_62809,630|HGLibA_62806,0
THADA	1895	3	HGLibA_49284,725|HGLibB_49225,927|HGLibA_49282,243|HGLibB_49224,0|HGLibA_49283,0|HGLibB_49223,0
hsa-mir-519a	1883	3	HGLibA_61711,963|HGLibA_61710,790|HGLibA_61709,130
SNAP29	1877	3	HGLibB_45827,1373|HGLibA_45880,283|HGLibB_45825,221|HGLibA_45882,0|HGLibB_45826,0|HGLibA_45881,0
TMEM165	1863	3	HGLibB_49975,6|HGLibB_49977,1229|HGLibA_50036,628|HGLibA_50035,0|HGLibB_49976,0|HGLibA_50037,0
MX1	1862	3	HGLibB_30562,1427|HGLibB_30561,350|HGLibB_30563,85|HGLibA_30603,0|HGLibA_30605,0|HGLibA_30604,0
NAIP	1862	3	HGLibB_31038,12|HGLibA_31082,1270|HGLibB_31039,580|HGLibA_31080,0|HGLibA_31081,0|HGLibB_31040,0
ALK	1861	3	HGLibB_01641,155|HGLibA_01642,1302|HGLibB_01642,404|HGLibA_01644,0|HGLibA_01643,0|HGLibB_01640,0
STAU1	1845	3	HGLibA_47404,1268|HGLibA_47406,192|HGLibB_47347,385|HGLibB_47349,0|HGLibB_47348,0|HGLibA_47405,0
FAM90A1	1829	3	HGLibA_16792,583|HGLibB_16769,1|HGLibA_16790,1245|HGLibB_16767,0|HGLibA_16791,0|HGLibB_16768,0
LANCL1	1827	3	HGLibA_26004,215|HGLibA_26003,446|HGLibB_25966,1166|HGLibB_25967,0|HGLibB_25968,0|HGLibA_26005,0
LRRC31	1819	3	HGLibA_27312,1637|HGLibB_27270,24|HGLibB_27272,158|HGLibB_27271,0|HGLibA_27310,0|HGLibA_27311,0
CACNA1C	1816	3	HGLibB_06876,45|HGLibB_06877,1727|HGLibB_06875,44|HGLibA_06881,0|HGLibA_06880,0|HGLibA_06882,0
SSBP2	1812	3	HGLibA_47118,1|HGLibB_47060,1147|HGLibB_47061,664|HGLibA_47117,0|HGLibB_47059,0|HGLibA_47116,0
MEGF9	1808	3	HGLibA_28859,180|HGLibB_28818,291|HGLibB_28817,1337|HGLibA_28858,0|HGLibB_28819,0|HGLibA_28860,0
SLC4A11	1803	3	HGLibA_45208,1747|HGLibA_45209,33|HGLibB_45154,23|HGLibB_45155,0|HGLibB_45156,0|HGLibA_45207,0
BMX	1780	3	HGLibA_04640,364|HGLibA_04642,1340|HGLibA_04641,76|HGLibB_04640,0|HGLibB_04638,0|HGLibB_04639,0
FARP2	1780	3	HGLibB_16855,1005|HGLibB_16854,22|HGLibB_16853,753|HGLibA_16878,0|HGLibA_16876,0|HGLibA_16877,0
REXO4	1768	3	HGLibB_40847,1236|HGLibA_40899,525|HGLibB_40848,7|HGLibB_40849,0|HGLibA_40900,0|HGLibA_40898,0
ITSN1	1762	3	HGLibB_23838,1123|HGLibA_23870,315|HGLibA_23872,324|HGLibB_23836,0|HGLibA_23871,0|HGLibB_23837,0
SUN1	1744	3	HGLibB_47730,292|HGLibB_47731,158|HGLibA_47788,1294|HGLibA_47787,0|HGLibB_47732,0|HGLibA_47789,0
ID4	1722	3	HGLibB_22502,378|HGLibA_22533,40|HGLibA_22532,1304|HGLibA_22534,0|HGLibB_22501,0|HGLibB_22503,0
FRS3	1715	3	HGLibB_18025,1447|HGLibB_18027,256|HGLibA_18050,12|HGLibA_18051,0|HGLibA_18049,0|HGLibB_18026,0
GPATCH8	1697	3	HGLibB_19722,615|HGLibB_19721,1045|HGLibB_19723,37|HGLibA_19751,0|HGLibA_19750,0|HGLibA_19749,0
C4BPB	1694	3	HGLibA_06294,1150|HGLibB_06289,322|HGLibB_06290,222|HGLibA_06292,0|HGLibA_06293,0|HGLibB_06288,0
ZNF24	1693	3	HGLibB_55590,352|HGLibB_55591,19|HGLibB_55589,1322|HGLibA_55657,0|HGLibA_55658,0|HGLibA_55656,0
LILRB1	1691	3	HGLibB_26471,3|HGLibA_26506,359|HGLibA_26507,1329|HGLibB_26470,0|HGLibB_26469,0|HGLibA_26508,0
S100A9	1690	3	HGLibA_42574,27|HGLibB_42522,1323|HGLibA_42575,340|HGLibB_42521,0|HGLibA_42576,0|HGLibB_42523,0
TFAP2A	1688	3	HGLibB_49061,21|HGLibB_49062,924|HGLibA_49121,743|HGLibB_49063,0|HGLibA_49122,0|HGLibA_49120,0

REXO2	1683	3	HGLibA_40897,3|HGLibA_40896,523|HGLibB_40844,1157|HGLibB_40846,0|HGLibB_40845,0|HGLibA_40895,0
LZTFL1	1672	3	HGLibB_27759,1262|HGLibB_27758,1|HGLibB_27760,409|HGLibB_27799,0|HGLibA_27800,0|HGLibA_27801,0
TBC1D10C	1657	3	HGLibB_48412,170|HGLibA_48469,10|HGLibB_48410,1477|HGLibB_48411,0|HGLibA_48471,0|HGLibA_48470,0
LAMP3	1655	3	HGLibB_25945,17|HGLibA_25983,603|HGLibA_25982,1035|HGLibB_25946,0|HGLibA_25984,0|HGLibB_25947,0
hsa-mir-8052	1654	3	HGLibA_64048,27|HGLibA_64045,1437|HGLibA_64047,190|HGLibA_64046,0
FHOD1	1651	3	HGLibB_17469,320|HGLibA_17492,1330|HGLibB_17470,1|HGLibB_17468,0|HGLibA_17493,0|HGLibA_17491,0
CHRD	1614	3	HGLibA_09534,1153|HGLibA_09535,61|HGLibA_09536,400|HGLibB_09525,0|HGLibB_09527,0|HGLibB_09526,0
GNL1	1605	3	HGLibB_19516,1298|HGLibB_19517,175|HGLibB_19518,132|HGLibA_19545,0|HGLibA_19544,0|HGLibA_19543,0
BIN3	1602	3	HGLibB_04509,37|HGLibB_04510,84|HGLibB_04511,1481|HGLibA_04511,0|HGLibA_04513,0|HGLibA_04512,0
H6PD	1600	3	HGLibB_20759,1347|HGLibA_20787,82|HGLibA_20786,171|HGLibB_20758,0|HGLibA_20788,0|HGLibB_20760,0
ARMC8	1593	3	HGLibB_03029,524|HGLibA_03031,1046|HGLibA_03032,23|HGLibB_03030,0|HGLibA_03033,0|HGLibB_03031,0
NRG3	1586	3	HGLibA_32683,86|HGLibA_32681,3|HGLibB_32637,1497|HGLibA_32682,0|HGLibB_32639,0|HGLibB_32638,0
SIRT5	1578	3	HGLibB_44175,1390|HGLibB_44174,1|HGLibA_44227,187|HGLibB_44176,0|HGLibA_44228,0|HGLibA_44229,0
LIPG	1576	3	HGLibB_26574,1325|HGLibA_26612,173|HGLibB_26575,78|HGLibA_26611,0|HGLibB_26576,0|HGLibA_26613,0
NXF5	1574	3	HGLibB_33093,22|HGLibA_33138,1066|HGLibB_33095,486|HGLibA_33137,0|HGLibB_33094,0|HGLibA_33139,0
COL1A1	1574	3	HGLibB_10518,316|HGLibA_10529,41|HGLibA_10531,1217|HGLibA_10530,0|HGLibB_10520,0|HGLibB_10519,0
NEURL1B	1573	3	HGLibB_31683,47|HGLibA_31725,1438|HGLibA_31726,88|HGLibB_31682,0|HGLibB_31681,0|HGLibA_31724,0
LCN9	1567	3	HGLibA_26186,181|HGLibB_26149,1096|HGLibB_26151,290|HGLibA_26187,0|HGLibB_26150,0|HGLibA_26188,0
SLC23A2	1556	3	HGLibA_44609,136|HGLibB_44556,77|HGLibB_44557,1343|HGLibB_44555,0|HGLibA_44610,0|HGLibA_44608,0
PLXND1	1556	3	HGLibA_37281,1437|HGLibA_37282,98|HGLibB_37232,21|HGLibA_37283,0|HGLibB_37233,0|HGLibB_37234,0
PATE3	1550	3	HGLibB_35230,69|HGLibB_35229,160|HGLibB_35231,1321|HGLibA_35277,0|HGLibA_35278,0|HGLibA_35276,0
BEND6	1548	3	HGLibB_04410,3|HGLibA_04413,171|HGLibA_04414,1374|HGLibB_04411,0|HGLibB_04412,0|HGLibA_04412,0
MED13	1543	3	HGLibA_28749,1|HGLibB_28708,438|HGLibB_28707,1104|HGLibB_28706,0|HGLibA_28747,0|HGLibA_28748,0
NSUN5	1537	3	HGLibB_32747,1124|HGLibA_32790,149|HGLibB_32746,264|HGLibB_32745,0|HGLibA_32791,0|HGLibA_32789,0
CES4A	1535	3	HGLibA_09228,1120|HGLibB_09220,287|HGLibB_09219,128|HGLibB_09218,0|HGLibA_09227,0|HGLibA_09226,0
OTOS	1530	3	HGLibA_34747,351|HGLibB_34701,14|HGLibA_34746,1165|HGLibB_34702,0|HGLibA_34748,0|HGLibB_34700,0
SNF8	1522	3	HGLibB_45871,103|HGLibB_45870,18|HGLibA_45927,1401|HGLibA_45925,0|HGLibA_45926,0|HGLibB_45869,0
SLITRK5	1518	3	HGLibA_45525,368|HGLibA_45524,1071|HGLibA_45526,79|HGLibB_45473,0|HGLibB_45472,0|HGLibB_45471,0
SLC13A3	1513	3	HGLibB_44338,39|HGLibB_44337,70|HGLibB_44336,1404|HGLibA_44389,0|HGLibA_44391,0|HGLibA_44390,0
MORC3	1500	3	HGLibA_29654,11|HGLibB_29614,1192|HGLibB_29613,297|HGLibA_29653,0|HGLibA_29655,0|HGLibB_29612,0
SEZ6L2	1493	3	HGLibB_43632,87|HGLibB_43633,687|HGLibA_43684,719|HGLibA_43686,0|HGLibB_43631,0|HGLibA_43685,0
IGBP1	1488	3	HGLibA_22783,1306|HGLibB_22750,181|HGLibA_22781,1|HGLibB_22752,0|HGLibB_22751,0|HGLibA_22782,0
UBB	1476	3	HGLibB_52498,1252|HGLibA_52565,63|HGLibB_52500,161|HGLibA_52564,0|HGLibB_52499,0|HGLibA_52563,0
CEP85L	1461	3	HGLibB_09164,75|HGLibB_09166,1365|HGLibB_09174,21|HGLibA_09173,0|HGLibA_09172,0|HGLibB_09165,0
RSPO2	1459	3	HGLibB_42293,47|HGLibA_42346,1409|HGLibB_42294,3|HGLibA_42347,0|HGLibA_42348,0|HGLibB_42295,0
MAN1A1	1457	3	HGLibA_28057,13|HGLibB_28018,91|HGLibB_28016,1353|HGLibA_28058,0|HGLibB_28017,0|HGLibA_28059,0
VRK2	1456	3	HGLibB_53761,151|HGLibB_53760,8|HGLibB_53762,1297|HGLibA_53828,0|HGLibA_53826,0|HGLibA_53827,0
GHR	1445	3	HGLibB_19014,1359|HGLibB_19015,83|HGLibB_19016,3|HGLibA_19040,0|HGLibA_19041,0|HGLibA_19039,0
APPL2	1431	3	HGLibB_02568,1348|HGLibB_02567,82|HGLibB_02566,1|HGLibA_02570,0|HGLibA_02568,0|HGLibA_02569,0
USP7	1429	3	HGLibB_53345,1238|HGLibB_53343,4|HGLibA_53410,187|HGLibA_53411,0|HGLibB_53344,0|HGLibA_53409,0
RIPK1	1421	3	HGLibB_41265,1313|HGLibA_41316,1|HGLibA_41315,107|HGLibB_41264,0|HGLibB_41263,0|HGLibA_41317,0
hsa-mir-320c	1418	3	HGLibA_58948,81|HGLibA_58946,1176|HGLibA_58949,161|HGLibA_58947,0
PBK	1412	3	HGLibB_35284,1276|HGLibA_35332,16|HGLibB_35283,120|HGLibA_35331,0|HGLibA_35330,0|HGLibB_35285,0
SEMA4A	1410	3	HGLibA_43340,1381|HGLibB_43286,3|HGLibB_43288,26|HGLibA_43339,0|HGLibA_43341,0|HGLibB_43287,0
ACSL5	1401	3	HGLibA_00562,17|HGLibB_00561,1379|HGLibA_00564,5|HGLibB_00562,0|HGLibB_00563,0|HGLibA_00563,0
COL24A1	1401	3	HGLibB_10538,2|HGLibB_10536,1|HGLibA_10548,1398|HGLibA_10547,0|HGLibB_10537,0|HGLibA_10549,0
RNF103	1399	3	HGLibA_41457,189|HGLibA_41458,1177|HGLibB_41406,33|HGLibB_41404,0|HGLibB_41405,0|HGLibA_41456,0
MYF6	1397	3	HGLibA_30700,664|HGLibB_30657,20|HGLibB_30659,713|HGLibB_30658,0|HGLibA_30701,0|HGLibA_30699,0
C1orf194	1396	3	HGLibA_05816,642|HGLibA_05815,2|HGLibB_05812,752|HGLibB_05811,0|HGLibA_05817,0|HGLibB_05813,0
PRMT2	1396	3	HGLibB_38573,1|HGLibA_38622,1313|HGLibB_38574,82|HGLibA_38623,0|HGLibB_38575,0|HGLibA_38624,0
PLK5	1394	3	HGLibB_37151,1281|HGLibB_37153,20|HGLibB_37152,93|HGLibA_37201,0|HGLibA_37199,0|HGLibA_37200,0
PPIAL4A	1392	3	HGLibA_37897,1|HGLibA_37898,1|HGLibB_37847,1390|HGLibB_37849,0|HGLibB_37848,0
RPGRIP1L	1391	3	HGLibB_41789,68|HGLibB_41787,38|HGLibA_41840,1285|HGLibB_41788,0|HGLibA_41842,0|HGLibA_41841,0
SOX7	1391	3	HGLibB_46217,1226|HGLibB_46218,2|HGLibA_46272,163|HGLibA_46274,0|HGLibB_46216,0|HGLibA_46273,0
SBSPON	1390	3	HGLibB_42763,1312|HGLibA_42814,12|HGLibA_42815,66|HGLibB_42761,0|HGLibA_42816,0|HGLibB_42762,0
RPL14	1382	3	HGLibA_41874,227|HGLibB_41822,588|HGLibB_41820,567|HGLibA_41873,0|HGLibB_41821,0|HGLibA_41875,0
TTLL11	1378	3	HGLibB_52127,178|HGLibB_52126,4|HGLibB_52128,1196|HGLibA_52193,0|HGLibA_52191,0|HGLibA_52192,0
ACBD3	1374	3	HGLibA_00398,30|HGLibB_00398,1257|HGLibB_00397,87|HGLibA_00397,0|HGLibA_00399,0|HGLibB_00399,0
EPPK1	1372	3	HGLibB_15341,1315|HGLibB_15339,15|HGLibA_15363,42|HGLibA_15362,0|HGLibA_15361,0|HGLibB_15340,0
CHST5	1370	3	HGLibA_09640,1281|HGLibB_09630,82|HGLibB_09631,7|HGLibA_09641,0|HGLibB_09632,0|HGLibA_09639,0
ACTRT1	1363	3	HGLibB_00677,4|HGLibA_00677,24|HGLibA_00676,1335|HGLibB_00675,0|HGLibA_00678,0|HGLibB_00676,0
B3GALT2	1360	3	HGLibA_03956,97|HGLibA_03958,1242|HGLibB_03955,21|HGLibB_03954,0|HGLibA_03957,0|HGLibB_03956,0
SLC27A6	1358	3	HGLibA_44828,10|HGLibB_44776,1291|HGLibB_44775,57|HGLibB_44774,0|HGLibA_44829,0|HGLibA_44827,0
AATK	1357	3	HGLibB_00088,29|HGLibA_00090,1327|HGLibB_00090,1|HGLibB_00089,0|HGLibA_00089,0|HGLibA_00088,0
PTOV1	1357	3	HGLibB_39351,1|HGLibA_39400,1310|HGLibB_39352,46|HGLibB_39350,0|HGLibA_39399,0|HGLibA_39401,0
ATP6V1H	1349	3	HGLibB_03763,23|HGLibB_03765,3|HGLibA_03767,1323|HGLibA_03766,0|HGLibB_03764,0|HGLibA_03765,0
MARK3	1346	3	HGLibB_28329,1206|HGLibB_28328,32|HGLibA_28369,108|HGLibA_28371,0|HGLibB_28330,0|HGLibA_28370,0
CSF1	1343	3	HGLibA_11321,34|HGLibA_11322,952|HGLibB_11309,357|HGLibB_11310,0|HGLibA_11320,0|HGLibB_11308,0
ATP6V1D	1342	3	HGLibB_03743,2|HGLibB_03742,51|HGLibA_03744,1289|HGLibB_03744,0|HGLibA_03746,0|HGLibA_03745,0
SLC25A33	1339	3	HGLibA_44712,8|HGLibA_44710,89|HGLibB_44659,1242|HGLibB_44657,0|HGLibA_44711,0|HGLibB_44658,0
PSG2	1334	3	HGLibB_38990,49|HGLibB_38991,1280|HGLibA_39040,5|HGLibA_39039,0|HGLibA_39041,0|HGLibB_38992,0
CBFB	1330	3	HGLibA_07390,1320|HGLibA_07392,9|HGLibB_07386,1|HGLibA_07391,0|HGLibB_07384,0|HGLibB_07385,0
NLRX1	1329	3	HGLibB_32097,597|HGLibB_32096,3|HGLibB_32095,729|HGLibA_32139,0|HGLibA_32138,0|HGLibA_32140,0
TNNI1	1324	3	HGLibB_50809,1276|HGLibB_50810,4|HGLibB_50808,44|HGLibA_50871,0|HGLibA_50870,0|HGLibA_50872,0
SCGB3A2	1319	3	HGLibA_42934,1302|HGLibB_42883,2|HGLibB_42881,15|HGLibA_42936,0|HGLibA_42935,0|HGLibB_42882,0
AP1M2	1304	3	HGLibA_02294,1|HGLibB_02290,1256|HGLibB_02291,47|HGLibB_02292,0|HGLibA_02292,0|HGLibA_02293,0
CDH12	1302	3	HGLibB_08630,5|HGLibB_08632,1280|HGLibB_08631,17|HGLibA_08639,0|HGLibA_08638,0|HGLibA_08637,0

<table>
<tr><td>SLC25A43</td><td>1301</td><td>3</td><td>HGLibB_44690,338|HGLibB_44691,891|HGLibA_44744,72|HGLibA_44745,0|HGLibB_44692,0|HGLibA_44743,0</td></tr>
<tr><td>NDN</td><td>1295</td><td>3</td><td>HGLibA_31398,8|HGLibB_31356,22|HGLibB_31355,1265|HGLibB_31357,0|HGLibA_31397,0|HGLibA_31399,0</td></tr>
<tr><td>ARF5</td><td>1284</td><td>3</td><td>HGLibB_02654,1136|HGLibB_02653,7|HGLibB_02652,141|HGLibA_02654,0|HGLibA_02655,0|HGLibA_02656,0</td></tr>
<tr><td>SYDE1</td><td>1280</td><td>3</td><td>HGLibB_47880,2|HGLibB_47882,673|HGLibA_47941,605|HGLibA_47939,0|HGLibA_47940,0|HGLibB_47881,0</td></tr>
<tr><td>NSUN3</td><td>1279</td><td>3</td><td>HGLibA_32785,3|HGLibB_32740,1275|HGLibA_32784,1|HGLibA_32783,0|HGLibB_32741,0|HGLibB_32739,0</td></tr>
<tr><td>ABI3BP</td><td>1279</td><td>3</td><td>HGLibA_00298,287|HGLibB_00299,6|HGLibB_00298,986|HGLibB_00300,0|HGLibA_00299,0|HGLibA_00300,0</td></tr>
<tr><td>PRMT3</td><td>1269</td><td>3</td><td>HGLibB_38577,951|HGLibA_38627,275|HGLibA_38625,43|HGLibA_38626,0|HGLibB_38576,0|HGLibB_38578,0</td></tr>
<tr><td>C10orf12</td><td>1267</td><td>3</td><td>HGLibB_05041,2|HGLibB_05040,1261|HGLibA_05043,4|HGLibA_05044,0|HGLibA_05042,0|HGLibB_05039,0</td></tr>
<tr><td>CRIP2</td><td>1261</td><td>3</td><td>HGLibB_11140,710|HGLibA_11154,4|HGLibB_11141,547|HGLibB_11142,0|HGLibA_11152,0|HGLibA_11153,0</td></tr>
<tr><td>PRH2</td><td>1257</td><td>3</td><td>HGLibA_38464,1097|HGLibA_38465,159|HGLibB_38414,1|HGLibA_38463,0|HGLibB_38415,0|HGLibB_38416,0</td></tr>
<tr><td>PROS1</td><td>1255</td><td>3</td><td>HGLibA_38690,6|HGLibB_38640,238|HGLibB_38641,1011|HGLibA_38688,0|HGLibA_38689,0|HGLibB_38639,0</td></tr>
<tr><td>RXFP1</td><td>1252</td><td>3</td><td>HGLibA_42493,1|HGLibB_42440,1|HGLibB_42441,1250|HGLibA_42494,0|HGLibB_42442,0|HGLibA_42495,0</td></tr>
<tr><td>CMBL</td><td>1250</td><td>3</td><td>HGLibA_10183,599|HGLibB_10172,648|HGLibA_10182,3|HGLibB_10173,0|HGLibA_10181,0|HGLibB_10171,0</td></tr>
<tr><td>RCAN3</td><td>1250</td><td>3</td><td>HGLibB_40622,1237|HGLibA_40674,9|HGLibB_40624,4|HGLibA_40673,0|HGLibA_40675,0|HGLibB_40623,0</td></tr>
<tr><td>AOAH</td><td>1231</td><td>3</td><td>HGLibB_02263,283|HGLibB_02262,1|HGLibA_02263,947|HGLibA_02265,0|HGLibA_02264,0|HGLibB_02261,0</td></tr>
<tr><td>PRDM8</td><td>1225</td><td>3</td><td>HGLibB_38352,1|HGLibB_38351,1181|HGLibA_38402,43|HGLibA_38401,0|HGLibA_38400,0|HGLibB_38353,0</td></tr>
<tr><td>ADPRHL1</td><td>1223</td><td>3</td><td>HGLibA_01029,2|HGLibB_01026,1139|HGLibA_01027,82|HGLibA_01028,0|HGLibB_01025,0|HGLibB_01027,0</td></tr>
<tr><td>CCDC23</td><td>1221</td><td>3</td><td>HGLibB_07728,64|HGLibB_07726,20|HGLibA_07734,1137|HGLibA_07732,0|HGLibB_07727,0|HGLibA_07733,0</td></tr>
<tr><td>RPA4</td><td>1219</td><td>3</td><td>HGLibA_41808,170|HGLibB_41756,2|HGLibB_41757,1047|HGLibB_41755,0|HGLibA_41807,0|HGLibA_41809,0</td></tr>
<tr><td>COPG2</td><td>1218</td><td>3</td><td>HGLibA_10707,1|HGLibB_10695,1091|HGLibB_10697,126|HGLibA_10708,0|HGLibB_10696,0|HGLibA_10706,0</td></tr>
<tr><td>UQCRH</td><td>1216</td><td>3</td><td>HGLibB_53057,1212|HGLibA_53123,2|HGLibA_53121,2|HGLibA_53122,0|HGLibB_53058,0|HGLibB_53056,0</td></tr>
<tr><td>JAK3</td><td>1211</td><td>3</td><td>HGLibA_23918,169|HGLibB_23885,1011|HGLibB_23886,31|HGLibB_23884,0|HGLibA_23920,0|HGLibA_23919,0</td></tr>
<tr><td>CEP170B</td><td>1205</td><td>3</td><td>HGLibB_09110,1200|HGLibA_09119,2|HGLibB_09111,3|HGLibA_09118,0|HGLibA_09120,0|HGLibB_09112,0</td></tr>
<tr><td>KCNC3</td><td>1203</td><td>3</td><td>HGLibB_24135,6|HGLibB_24134,1196|HGLibB_24133,1|HGLibA_24167,0|HGLibA_24168,0|HGLibA_24169,0</td></tr>
<tr><td>SPATA31D1</td><td>1192</td><td>3</td><td>HGLibA_46495,29|HGLibB_46439,1133|HGLibB_46438,30|HGLibB_46440,0|HGLibA_46496,0|HGLibA_46494,0</td></tr>
<tr><td>OR2A1</td><td>1188</td><td>3</td><td>HGLibB_33673,8|HGLibA_33718,6|HGLibA_33719,1174|HGLibA_33720,0|HGLibB_33674,0|HGLibB_33675,0</td></tr>
<tr><td>N4BP3</td><td>1185</td><td>3</td><td>HGLibA_30961,1131|HGLibB_30918,2|HGLibB_30920,52|HGLibA_30960,0|HGLibB_30919,0|HGLibA_30962,0</td></tr>
<tr><td>SH3RF1</td><td>1182</td><td>3</td><td>HGLibA_43969,68|HGLibB_43917,80|HGLibA_43971,1034|HGLibA_43970,0|HGLibB_43916,0|HGLibB_43918,0</td></tr>
<tr><td>CCDC25</td><td>1172</td><td>3</td><td>HGLibB_07733,19|HGLibB_07732,1148|HGLibA_07739,5|HGLibA_07738,0|HGLibA_07740,0|HGLibB_07734,0</td></tr>
<tr><td>HMGA2</td><td>1125</td><td>3</td><td>HGLibB_21658,587|HGLibB_21659,110|HGLibA_21686,428|HGLibB_21660,0|HGLibA_21688,0|HGLibA_21687,0</td></tr>
<tr><td>AGPAT9</td><td>1123</td><td>3</td><td>HGLibB_01232,1096|HGLibB_01234,23|HGLibB_01233,4|HGLibA_01234,0|HGLibA_01236,0|HGLibA_01235,0</td></tr>
<tr><td>PPHLN1</td><td>1101</td><td>3</td><td>HGLibB_37841,4|HGLibA_37890,732|HGLibB_37843,365|HGLibA_37891,0|HGLibB_37842,0|HGLibA_37892,0</td></tr>
<tr><td>RPL7A</td><td>1098</td><td>3</td><td>HGLibA_41995,774|HGLibA_41996,40|HGLibB_41944,284|HGLibB_41943,0|HGLibA_41997,0|HGLibB_41942,0</td></tr>
<tr><td>PM20D2</td><td>1086</td><td>3</td><td>HGLibA_37288,6|HGLibB_37238,1079|HGLibA_37289,1|HGLibA_37287,0|HGLibB_37239,0|HGLibB_37240,0</td></tr>
<tr><td>RSC1A1</td><td>1076</td><td>3</td><td>HGLibA_42308,1071|HGLibA_42307,1|HGLibB_42256,4|HGLibB_42254,0|HGLibB_42255,0|HGLibA_42309,0</td></tr>
<tr><td>OR2T1</td><td>1063</td><td>3</td><td>HGLibB_33807,15|HGLibB_33805,1047|HGLibA_33850,1|HGLibB_33806,0|HGLibA_33852,0|HGLibA_33851,0</td></tr>
<tr><td>GRK4</td><td>1049</td><td>3</td><td>HGLibB_20302,381|HGLibB_20301,665|HGLibA_20328,3|HGLibA_20329,0|HGLibB_20300,0|HGLibA_20330,0</td></tr>
<tr><td>PATE1</td><td>1041</td><td>3</td><td>HGLibA_35270,12|HGLibB_35223,7|HGLibA_35271,1022|HGLibA_35272,0|HGLibB_35225,0|HGLibB_35224,0</td></tr>
<tr><td>CCT2</td><td>1039</td><td>3</td><td>HGLibB_08189,116|HGLibB_08188,20|HGLibA_08196,903|HGLibB_08187,0|HGLibA_08194,0|HGLibA_08195,0</td></tr>
<tr><td>CGA</td><td>1038</td><td>3</td><td>HGLibA_09295,865|HGLibA_09296,133|HGLibB_09288,40|HGLibB_09287,0|HGLibB_09289,0|HGLibA_09297,0</td></tr>
<tr><td>RAP1A</td><td>1015</td><td>3</td><td>HGLibA_40221,688|HGLibA_40220,317|HGLibA_40222,10|HGLibB_40169,0|HGLibB_40170,0|HGLibB_40171,0</td></tr>
<tr><td>PLP1</td><td>988</td><td>3</td><td>HGLibA_37217,15|HGLibB_37168,856|HGLibB_37167,117|HGLibA_37215,0|HGLibB_37166,0|HGLibA_37216,0</td></tr>
<tr><td>TIMP2</td><td>979</td><td>3</td><td>HGLibA_49539,299|HGLibA_49537,124|HGLibB_49479,556|HGLibB_49478,0|HGLibB_49477,0|HGLibA_49538,0</td></tr>
<tr><td>INPP5E</td><td>979</td><td>3</td><td>HGLibA_23352,89|HGLibB_23319,253|HGLibB_23320,637|HGLibA_23353,0|HGLibB_23321,0|HGLibA_23351,0</td></tr>
<tr><td>SLC25A18</td><td>963</td><td>3</td><td>HGLibA_44659,39|HGLibB_44607,742|HGLibB_44608,182|HGLibB_44606,0|HGLibA_44661,0|HGLibA_44660,0</td></tr>
<tr><td>MED28</td><td>963</td><td>3</td><td>HGLibB_28755,654|HGLibB_28754,137|HGLibA_28795,172|HGLibA_28797,0|HGLibA_28796,0|HGLibB_28756,0</td></tr>
<tr><td>MAL2</td><td>957</td><td>3</td><td>HGLibA_28024,321|HGLibB_27984,625|HGLibA_28026,11|HGLibB_27985,0|HGLibB_27983,0|HGLibA_28025,0</td></tr>
<tr><td>FBXO33</td><td>956</td><td>3</td><td>HGLibB_17051,403|HGLibA_17075,6|HGLibB_17052,547|HGLibA_17076,0|HGLibB_17053,0|HGLibA_17074,0</td></tr>
<tr><td>MEOX2</td><td>955</td><td>3</td><td>HGLibB_28851,269|HGLibA_28893,253|HGLibB_28850,433|HGLibA_28891,0|HGLibB_28852,0|HGLibA_28892,0</td></tr>
<tr><td>C1orf159</td><td>950</td><td>3</td><td>HGLibB_05780,286|HGLibB_05778,657|HGLibA_05782,7|HGLibA_05783,0|HGLibB_05779,0|HGLibA_05784,0</td></tr>
<tr><td>IL4I1</td><td>942</td><td>3</td><td>HGLibB_23138,939|HGLibA_23168,2|HGLibA_23170,1|HGLibA_23169,0|HGLibB_23137,0|HGLibB_23139,0</td></tr>
<tr><td>PNLIPRP2</td><td>928</td><td>3</td><td>HGLibA_37359,38|HGLibA_37361,196|HGLibB_37312,694|HGLibA_37360,0|HGLibB_37311,0|HGLibB_37310,0</td></tr>
<tr><td>TGIF2LY</td><td>916</td><td>3</td><td>HGLibB_49192,20|HGLibB_49190,175|HGLibA_49251,721|HGLibA_49250,0|HGLibA_49249,0|HGLibB_49191,0</td></tr>
<tr><td>MYH1</td><td>907</td><td>3</td><td>HGLibB_30662,1|HGLibB_30661,714|HGLibA_30704,192|HGLibA_30703,0|HGLibA_30702,0|HGLibB_30660,0</td></tr>
<tr><td>GTPBP8</td><td>906</td><td>3</td><td>HGLibA_20622,323|HGLibB_20593,582|HGLibB_20594,1|HGLibA_20623,0|HGLibB_20595,0|HGLibA_20621,0</td></tr>
<tr><td>SVIL</td><td>904</td><td>3</td><td>HGLibA_47886,568|HGLibA_47885,30|HGLibB_47828,306|HGLibB_47829,0|HGLibB_47830,0|HGLibA_47887,0</td></tr>
<tr><td>BECN1</td><td>902</td><td>3</td><td>HGLibA_04395,3|HGLibA_04394,897|HGLibB_04394,2|HGLibB_04392,0|HGLibB_04393,0|HGLibA_04396,0</td></tr>
<tr><td>NPY2R</td><td>901</td><td>3</td><td>HGLibB_32510,164|HGLibA_32552,74|HGLibB_32509,663|HGLibA_32553,0|HGLibB_32508,0|HGLibA_32554,0</td></tr>
<tr><td>STK19</td><td>879</td><td>3</td><td>HGLibA_47469,215|HGLibA_47468,578|HGLibB_47410,86|HGLibA_47467,0|HGLibB_47412,0|HGLibB_47411,0</td></tr>
<tr><td>ASIP</td><td>877</td><td>3</td><td>HGLibB_03313,518|HGLibB_03311,4|HGLibA_03314,355|HGLibA_03313,0|HGLibA_03315,0|HGLibB_03312,0</td></tr>
<tr><td>ADRA2C</td><td>876</td><td>3</td><td>HGLibA_01052,665|HGLibA_01053,41|HGLibB_01050,170|HGLibB_01051,0|HGLibA_01051,0|HGLibB_01049,0</td></tr>
<tr><td>INTS2</td><td>871</td><td>3</td><td>HGLibA_23415,1|HGLibB_23383,788|HGLibA_23414,82|HGLibB_23384,0|HGLibB_23382,0|HGLibA_23416,0</td></tr>
<tr><td>CEP89</td><td>868</td><td>3</td><td>HGLibA_09177,179|HGLibB_09167,10|HGLibB_09169,679|HGLibA_09176,0|HGLibB_09168,0|HGLibA_09175,0</td></tr>
<tr><td>SLC9A9</td><td>860</td><td>3</td><td>HGLibA_45429,843|HGLibB_45377,6|HGLibB_45376,11|HGLibA_45430,0|HGLibA_45431,0|HGLibB_45378,0</td></tr>
<tr><td>ACSM2B</td><td>825</td><td>3</td><td>HGLibA_00576,1|HGLibB_00573,33|HGLibB_00574,791|HGLibA_00574,0|HGLibA_00575,0|HGLibB_00575,0</td></tr>
<tr><td>PYCR2</td><td>824</td><td>3</td><td>HGLibA_39675,646|HGLibA_39677,1|HGLibB_39625,177|HGLibB_39624,0|HGLibB_39626,0|HGLibA_39676,0</td></tr>
<tr><td>MAD2L1BP</td><td>820</td><td>3</td><td>HGLibB_27806,2|HGLibA_27848,645|HGLibA_27849,173|HGLibA_27847,0|HGLibB_27808,0|HGLibB_27807,0</td></tr>
<tr><td>PLA2G16</td><td>814</td><td>3</td><td>HGLibA_36878,90|HGLibB_36832,684|HGLibA_36879,40|HGLibB_36833,0|HGLibA_36880,0|HGLibB_36831,0</td></tr>
<tr><td>KIAA1430</td><td>813</td><td>3</td><td>HGLibB_24721,79|HGLibB_24720,58|HGLibB_24719,676|HGLibA_24753,0|HGLibA_24755,0|HGLibA_24754,0</td></tr>
<tr><td>RGL2</td><td>808</td><td>3</td><td>HGLibA_41005,788|HGLibB_40953,1|HGLibB_40952,19|HGLibA_41003,0|HGLibB_40954,0|HGLibA_41004,0</td></tr>
<tr><td>OR5D18</td><td>792</td><td>3</td><td>HGLibA_34283,342|HGLibA_34284,89|HGLibB_34237,361|HGLibB_34239,0|HGLibB_34238,0|HGLibA_34282,0</td></tr>
<tr><td>TOR4A</td><td>783</td><td>3</td><td>HGLibB_50969,690|HGLibB_50971,82|HGLibB_50970,11|HGLibA_51034,0|HGLibA_51032,0|HGLibA_51033,0</td></tr>
<tr><td>RASA1</td><td>781</td><td>3</td><td>HGLibB_40244,1|HGLibB_40245,35|HGLibA_40297,745|HGLibA_40295,0|HGLibA_40296,0|HGLibB_40246,0</td></tr>
<tr><td>OR10G7</td><td>779</td><td>3</td><td>HGLibA_33497,189|HGLibB_33453,4|HGLibB_33451,586|HGLibB_33452,0|HGLibA_33498,0|HGLibA_33496,0</td></tr>
<tr><td>VSIG8</td><td>769</td><td>3</td><td>HGLibB_53786,99|HGLibB_53784,3|HGLibA_53851,667|HGLibB_53785,0|HGLibA_53852,0|HGLibA_53850,0</td></tr>
<tr><td>CAPN12</td><td>763</td><td>3</td><td>HGLibB_07145,8|HGLibA_07152,577|HGLibA_07151,178|HGLibB_07147,0|HGLibA_07150,0|HGLibB_07146,0</td></tr>
<tr><td>MEOX1</td><td>759</td><td>3</td><td>HGLibB_28848,3|HGLibB_28847,740|HGLibA_28888,16|HGLibA_28889,0|HGLibA_28890,0|HGLibB_28849,0</td></tr>
</table>

NDUFB9	753	3	HGLibB_31492,307|HGLibB_31490,2|HGLibA_31533,444|HGLibA_31534,0|HGLibB_31491,0|HGLibA_31532,0
PARVB	743	3	HGLibB_35212,722|HGLibA_35260,1|HGLibB_35211,20|HGLibB_35213,0|HGLibA_35259,0|HGLibA_35258,0
ZNF331	740	3	HGLibA_55817,2|HGLibB_55748,373|HGLibA_55815,365|HGLibB_55749,0|HGLibB_55750,0|HGLibA_55816,0
C1QTNF9B	735	3	HGLibA_05964,2|HGLibB_05959,2|HGLibB_05960,731|HGLibA_05963,0|HGLibA_05962,0|HGLibB_05958,0
CRY1	734	3	HGLibA_11240,39|HGLibB_11229,685|HGLibA_11239,10|HGLibB_11228,0|HGLibB_11227,0|HGLibA_11241,0
ATP13A4	732	3	HGLibB_03572,12|HGLibA_03574,528|HGLibB_03574,192|HGLibB_03573,0|HGLibA_03576,0|HGLibA_03575,0
DNAJB2	732	3	HGLibB_13519,631|HGLibB_13517,76|HGLibA_13538,25|HGLibB_13518,0|HGLibA_13536,0|HGLibA_13537,0
TTLL10	731	3	HGLibA_52190,83|HGLibB_52123,618|HGLibB_52125,30|HGLibA_52188,0|HGLibA_52189,0|HGLibB_52124,0
CIAO1	725	3	HGLibB_09668,41|HGLibB_09666,681|HGLibA_09675,3|HGLibB_09667,0|HGLibA_09676,0|HGLibA_09677,0
SCARB1	724	3	HGLibB_42817,84|HGLibA_42870,638|HGLibB_42816,2|HGLibB_42815,0|HGLibA_42869,0|HGLibA_42868,0
GPRC5A	721	3	HGLibB_20098,2|HGLibB_20096,2|HGLibA_20125,717|HGLibB_20097,0|HGLibA_20126,0|HGLibA_20124,0
ITIH6	717	3	HGLibA_23817,677|HGLibB_23784,4|HGLibA_23816,36|HGLibA_23818,0|HGLibB_23783,0|HGLibB_23782,0
FAM83E	713	3	HGLibB_16730,661|HGLibB_16729,7|HGLibB_16728,45|HGLibA_16751,0|HGLibA_16753,0|HGLibA_16752,0
hsa-mir-4781	711	3	HGLibA_61233,20|HGLibA_61231,47|HGLibA_61230,644|HGLibA_61232,0
MAGEA11	702	3	HGLibB_27859,134|HGLibB_27857,564|HGLibA_27900,4|HGLibA_27898,0|HGLibA_27899,0|HGLibB_27858,0
PGM2	700	3	HGLibA_36263,670|HGLibB_36217,1|HGLibA_36264,29|HGLibA_36265,0|HGLibB_36216,0|HGLibB_36218,0
RNF5	698	3	HGLibB_41639,3|HGLibA_41690,1|HGLibB_41638,694|HGLibA_41692,0|HGLibA_41691,0|HGLibB_41640,0
RHEBL1	691	3	HGLibB_41107,651|HGLibA_41157,18|HGLibA_41156,22|HGLibA_41158,0|HGLibB_41105,0|HGLibB_41106,0
THRA	685	3	HGLibA_49389,674|HGLibB_49328,1|HGLibB_49329,10|HGLibB_49327,0|HGLibA_49388,0|HGLibA_49387,0
PRSS35	684	3	HGLibA_38928,8|HGLibA_38929,626|HGLibA_38930,50|HGLibB_38880,0|HGLibB_38879,0|HGLibB_38881,0
UNC5B	684	3	HGLibB_52953,536|HGLibA_53018,72|HGLibB_52952,76|HGLibA_53016,0|HGLibA_53017,0|HGLibB_52951,0
DCAF15	681	3	HGLibA_12430,1|HGLibA_12431,1|HGLibB_12417,679|HGLibA_12429,0|HGLibB_12415,0|HGLibB_12416,0
CHIT1	678	3	HGLibA_09454,663|HGLibA_09453,1|HGLibB_09446,14|HGLibB_09444,0|HGLibA_09455,0|HGLibB_09445,0
TMEM225	670	3	HGLibA_50245,1|HGLibA_50247,1|HGLibB_50187,668|HGLibB_50186,0|HGLibB_50185,0|HGLibA_50246,0
HUS1B	670	3	HGLibA_22429,668|HGLibA_22428,1|HGLibB_22398,1|HGLibA_22427,0|HGLibB_22396,0|HGLibB_22397,0
RASAL1	669	3	HGLibB_40260,657|HGLibA_40312,8|HGLibA_40311,4|HGLibB_40259,0|HGLibA_40310,0|HGLibB_40261,0
GIMAP7	667	3	HGLibB_19064,654|HGLibB_19062,3|HGLibA_19088,10|HGLibA_19087,0|HGLibB_19063,0|HGLibA_19089,0
UBE2G1	648	3	HGLibA_52605,2|HGLibB_52540,512|HGLibB_52542,134|HGLibA_52607,0|HGLibB_52541,0|HGLibA_52606,0
PRSS36	642	3	HGLibB_38884,24|HGLibA_38933,9|HGLibB_38883,609|HGLibB_38882,0|HGLibA_38932,0|HGLibA_38931,0
SCUBE2	641	3	HGLibB_43020,371|HGLibA_43073,30|HGLibB_43019,240|HGLibA_43072,0|HGLibA_43074,0|HGLibB_43021,0
TMEM132E	604	3	HGLibB_49881,28|HGLibA_49939,26|HGLibB_49879,550|HGLibA_49941,0|HGLibA_49940,0|HGLibB_49880,0
RAI1	598	3	HGLibA_40139,39|HGLibB_40088,532|HGLibB_40086,27|HGLibB_40087,0|HGLibA_40137,0|HGLibA_40138,0
HTR1A	582	3	HGLibB_22325,2|HGLibA_22355,579|HGLibB_22326,1|HGLibA_22357,0|HGLibA_22356,0|HGLibB_22324,0
PRAMEF15	578	3	HGLibA_38293,1|HGLibB_38245,573|HGLibA_38292,4|HGLibB_38243,0|HGLibB_38244,0
ZC2HC1B	566	3	HGLibB_54873,83|HGLibA_54939,3|HGLibA_54940,480|HGLibA_54941,0|HGLibB_54872,0|HGLibB_54874,0
FASN	527	3	HGLibB_16871,203|HGLibB_16873,1|HGLibA_16894,323|HGLibB_16872,0|HGLibA_16895,0|HGLibA_16896,0
HDGFRP3	521	3	HGLibA_21047,500|HGLibB_21019,18|HGLibA_21048,3|HGLibB_21021,0|HGLibA_21049,0|HGLibB_21020,0
BSCL2	513	3	HGLibB_04859,171|HGLibA_04859,17|HGLibB_04858,325|HGLibA_04860,0|HGLibA_04861,0|HGLibB_04857,0
EFS	500	3	HGLibA_14613,352|HGLibB_14589,1|HGLibA_14611,147|HGLibB_14590,0|HGLibB_14591,0|HGLibA_14612,0
LAIR1	496	3	HGLibB_25896,15|HGLibA_25931,414|HGLibB_25895,67|HGLibA_25932,0|HGLibB_25894,0|HGLibA_25933,0
FHL1	494	3	HGLibB_17457,355|HGLibA_17481,122|HGLibA_17480,17|HGLibA_17479,0|HGLibB_17456,0|HGLibB_17458,0
MOCS2	463	3	HGLibA_29614,25|HGLibB_29573,437|HGLibA_29616,1|HGLibB_29575,0|HGLibB_29574,0|HGLibA_29615,0
SAA2-SAA4	463	3	HGLibB_42561,23|HGLibA_42615,80|HGLibA_42614,360
SERTAD4	459	3	HGLibB_43561,155|HGLibB_43559,88|HGLibA_43614,216|HGLibB_43560,0|HGLibA_43613,0|HGLibA_43612,0
ETF1	457	3	HGLibB_15596,77|HGLibB_15594,209|HGLibA_15617,171|HGLibB_15595,0|HGLibA_15616,0|HGLibA_15618,0
MOGAT3	441	3	HGLibA_29631,2|HGLibA_29629,298|HGLibB_29590,141|HGLibA_29630,0|HGLibB_29588,0|HGLibB_29589,0
OXA1L	430	3	HGLibB_34763,361|HGLibB_34765,62|HGLibA_34809,7|HGLibA_34811,0|HGLibA_34810,0|HGLibB_34764,0
SEC31A	429	3	HGLibB_43187,401|HGLibA_43240,9|HGLibB_43188,19|HGLibB_43189,0|HGLibA_43241,0|HGLibA_43242,0
SFTPD	427	3	HGLibB_43726,35|HGLibB_43724,16|HGLibB_43725,376|HGLibA_43778,0|HGLibA_43777,0|HGLibA_43779,0
SPON2	420	3	HGLibA_46761,4|HGLibA_46759,336|HGLibB_46702,80|HGLibA_46760,0|HGLibB_46704,0|HGLibB_46703,0
AMH	417	3	HGLibB_01782,4|HGLibB_01783,1|HGLibA_01785,412|HGLibB_01781,0|HGLibA_01784,0|HGLibA_01783,0
CDK5RAP1	410	3	HGLibB_08775,150|HGLibA_08781,39|HGLibB_08776,221|HGLibB_08774,0|HGLibA_08783,0|HGLibA_08782,0
MYRF	410	3	HGLibB_30875,141|HGLibB_30873,26|HGLibB_30874,243|HGLibA_30915,0|HGLibA_30917,0|HGLibA_30916,0
OR1M1	404	3	HGLibA_33700,1|HGLibB_33655,48|HGLibB_33656,355|HGLibA_33701,0|HGLibB_33657,0|HGLibA_33702,0
PRG3	400	3	HGLibB_38407,348|HGLibA_38455,2|HGLibB_38405,50|HGLibA_38454,0|HGLibA_38456,0|HGLibB_38406,0
NTHL1	379	3	HGLibA_32845,4|HGLibB_32801,1|HGLibA_32844,374|HGLibB_32800,0|HGLibB_32799,0|HGLibA_32843,0
BMP8B	373	3	HGLibB_04620,2|HGLibA_04622,356|HGLibB_04621,15|HGLibB_04622,0|HGLibA_04623,0
HSD17B12	365	3	HGLibA_22177,337|HGLibB_22148,25|HGLibB_22149,3|HGLibA_22179,0|HGLibB_22150,0|HGLibA_22178,0
CRHR2	356	3	HGLibA_11144,14|HGLibA_11145,10|HGLibB_11133,332|HGLibA_11143,0|HGLibB_11131,0|HGLibB_11132,0
BMP3	347	3	HGLibB_04603,245|HGLibA_04606,7|HGLibB_04604,95|HGLibA_04604,0|HGLibB_04602,0|HGLibA_04605,0
ZNF337	338	3	HGLibB_55760,287|HGLibA_55827,11|HGLibB_55761,40|HGLibB_55762,0|HGLibA_55828,0|HGLibA_55829,0
C4orf17	334	3	HGLibB_06293,2|HGLibB_06291,331|HGLibA_06297,1|HGLibA_06295,0|HGLibB_06292,0|HGLibA_06296,0
THAP6	334	3	HGLibA_49308,3|HGLibB_49248,280|HGLibA_49306,51|HGLibA_49307,0|HGLibB_49247,0|HGLibB_49249,0
OCM2	326	3	HGLibB_33216,320|HGLibB_33217,2|HGLibB_33215,4|HGLibA_33261,0|HGLibA_33259,0|HGLibA_33260,0
DIP2A	325	3	HGLibB_13230,1|HGLibB_13228,2|HGLibA_13247,322|HGLibB_13229,0|HGLibA_13248,0|HGLibA_13246,0
hsa-mir-5003	321	3	HGLibA_61419,73|HGLibA_61420,242|HGLibA_61418,6|HGLibA_61421,0
MARVELD3	318	3	HGLibA_28385,303|HGLibB_28343,10|HGLibB_28344,5|HGLibA_28384,0|HGLibA_28386,0|HGLibB_28345,0
CARD8	316	3	HGLibB_07233,1|HGLibB_07232,151|HGLibA_07239,164|HGLibB_07231,0|HGLibA_07237,0|HGLibA_07238,0
hsa-mir-4757	282	3	HGLibA_61126,189|HGLibA_61124,12|HGLibA_61125,81|HGLibA_61127,0
PGF	282	3	HGLibB_36188,175|HGLibB_36186,23|HGLibB_36187,84|HGLibA_36233,0|HGLibA_36235,0|HGLibA_36234,0
SUMF1	271	3	HGLibA_47769,62|HGLibA_47770,205|HGLibB_47713,4|HGLibA_47771,0|HGLibB_47714,0|HGLibB_47712,0
GNAT1	257	3	HGLibB_19446,3|HGLibB_19445,91|HGLibB_19447,163|HGLibA_19471,0|HGLibA_19472,0|HGLibA_19473,0
PRRG1	239	3	HGLibA_38869,173|HGLibA_38868,20|HGLibB_38819,46|HGLibA_38870,0|HGLibB_38820,0|HGLibB_38821,0
KCNB2	233	3	HGLibA_24159,169|HGLibA_24158,43|HGLibB_24125,21|HGLibB_24126,0|HGLibB_24124,0|HGLibA_24160,0
RANBP1	232	3	HGLibB_40141,50|HGLibB_40140,20|HGLibA_40193,162|HGLibA_40192,0|HGLibA_40191,0|HGLibB_40142,0
SLC7A7	227	3	HGLibA_45380,7|HGLibA_45379,172|HGLibB_45326,48|HGLibA_45378,0|HGLibB_45325,0|HGLibB_45327,0
SLC4A1	227	3	HGLibB_45149,79|HGLibA_45201,124|HGLibB_45148,24|HGLibA_45203,0|HGLibB_45150,0|HGLibA_45202,0

WDR41	225	3	HGLibA_54087,1|HGLibB_54023,223|HGLibB_54022,1|HGLibA_54089,0|HGLibB_54021,0|HGLibA_54088,0
BPHL	223	3	HGLibA_04703,6|HGLibB_04702,169|HGLibB_04703,48|HGLibB_04701,0|HGLibA_04704,0|HGLibA_04705,0
ARIH1	209	3	HGLibB_02911,25|HGLibB_02909,138|HGLibB_02910,46|HGLibA_02911,0|HGLibA_02912,0|HGLibA_02913,0
FOXA1	198	3	HGLibB_17784,161|HGLibA_17806,13|HGLibB_17783,24|HGLibB_17785,0|HGLibA_17807,0|HGLibA_17808,0
CD177	192	3	HGLibB_08248,6|HGLibA_08255,179|HGLibA_08254,7|HGLibA_08256,0|HGLibB_08249,0|HGLibB_08247,0
TMEM120A	188	3	HGLibB_49836,37|HGLibB_49835,89|HGLibA_49896,62|HGLibA_49895,0|HGLibB_49834,0|HGLibA_49894,0
NDP	187	3	HGLibB_31368,39|HGLibB_31367,106|HGLibA_31409,42|HGLibA_31410,0|HGLibA_31411,0|HGLibB_31369,0
TSFM	185	3	HGLibB_51776,3|HGLibA_51841,21|HGLibB_51775,161|HGLibB_51777,0|HGLibA_51840,0|HGLibA_51842,0
TK2	180	3	HGLibA_49582,1|HGLibA_49583,174|HGLibB_49522,5|HGLibB_49524,0|HGLibB_49523,0|HGLibA_49584,0
SPRR2F	178	3	HGLibA_46819,1|HGLibA_46820,176|HGLibB_46762,1|HGLibA_46821,0
IQCF1	168	3	HGLibB_23470,74|HGLibB_23469,76|HGLibB_23468,18|HGLibA_23502,0|HGLibA_23501,0|HGLibA_23503,0
CARF	165	3	HGLibA_07243,127|HGLibB_07237,6|HGLibA_07244,32|HGLibA_07245,0|HGLibB_07238,0|HGLibB_07239,0
TMPRSS3	163	3	HGLibA_50638,2|HGLibB_50580,77|HGLibB_50579,84|HGLibA_50640,0|HGLibA_50639,0|HGLibB_50578,0
TSSK4	153	3	HGLibA_52019,34|HGLibB_51952,44|HGLibB_51953,75|HGLibA_52018,0|HGLibA_52017,0|HGLibB_51954,0
ZNF474	145	3	HGLibB_55994,18|HGLibB_55995,1|HGLibA_56061,126|HGLibA_56062,0|HGLibB_55996,0|HGLibA_56063,0
ARAP3	125	3	HGLibB_02630,76|HGLibA_02631,1|HGLibA_02632,48|HGLibB_02629,0|HGLibB_02628,0|HGLibA_02630,0
LTF	124	3	HGLibA_27609,67|HGLibB_27567,55|HGLibB_27568,2|HGLibA_27608,0|HGLibB_27569,0|HGLibA_27607,0
CD97	121	3	HGLibB_08480,6|HGLibB_08479,44|HGLibB_08478,71|HGLibA_08486,0|HGLibA_08485,0|HGLibA_08487,0
MEN1	120	3	HGLibB_28844,22|HGLibB_28846,6|HGLibA_28886,92|HGLibA_28885,0|HGLibA_28887,0|HGLibB_28845,0
NEGR1	115	3	HGLibA_31627,103|HGLibA_31625,10|HGLibB_31585,2|HGLibB_31583,0|HGLibA_31626,0|HGLibB_31584,0
KIAA1549L	107	3	HGLibB_24748,2|HGLibB_24746,25|HGLibA_24780,80|HGLibA_24781,0|HGLibA_24782,0|HGLibB_24747,0
TOP2B	105	3	HGLibA_50996,80|HGLibB_50933,1|HGLibB_50934,24|HGLibA_50998,0|HGLibB_50935,0|HGLibA_50997,0
ZNF177	86	3	HGLibB_55464,3|HGLibB_55465,1|HGLibA_55532,82|HGLibA_55531,0|HGLibA_55530,0
CT47B1	82	3	HGLibA_11552,78|HGLibB_11540,3|HGLibA_11551,1|HGLibB_11541,0
PATE4	82	3	HGLibB_35233,3|HGLibB_35234,1|HGLibB_35232,78|HGLibA_35281,0|HGLibA_35279,0|HGLibA_35280,0
OR2T3	78	3	HGLibA_33873,72|HGLibB_33826,1|HGLibB_33828,5|HGLibB_33827,0|HGLibA_33872,0|HGLibA_33871,0
CHCHD4	74	3	HGLibA_09371,37|HGLibA_09370,35|HGLibA_09372,2|HGLibB_09363,0|HGLibB_09364,0|HGLibB_09362,0
UBIAD1	36	3	HGLibA_52698,23|HGLibB_52634,4|HGLibB_52635,9|HGLibA_52700,0|HGLibB_52633,0|HGLibA_52699,0
COPB1	34	3	HGLibB_10683,30|HGLibB_10684,3|HGLibB_10685,1|HGLibA_10695,0|HGLibA_10696,0|HGLibA_10694,0
CNGA4	27	3	HGLibA_10272,1|HGLibB_10260,14|HGLibB_10261,12|HGLibA_10273,0|HGLibB_10262,0|HGLibA_10271,0
QPCT	23	3	HGLibA_39727,4|HGLibB_39677,18|HGLibB_39675,1|HGLibB_39676,0|HGLibA_39728,0|HGLibA_39726,0
SHANK1	22	3	HGLibB_43935,3|HGLibA_43987,13|HGLibB_43936,6|HGLibA_43989,0|HGLibA_43988,0|HGLibB_43934,0
NT5DC3	22	3	HGLibB_32781,5|HGLibB_32783,16|HGLibA_32827,1|HGLibB_32782,0|HGLibA_32826,0|HGLibA_32825,0
TUSC5	20	3	HGLibA_52351,18|HGLibA_52352,1|HGLibB_52287,1|HGLibB_52286,0|HGLibB_52285,0|HGLibA_52350,0
MRPL38	4	3	HGLibB_29927,1|HGLibB_29928,2|HGLibA_29968,1|HGLibA_29970,0|HGLibB_29929,0|HGLibA_29969,0
