# Supplementary material for: CRISPR/Cas9‐mediated whole genomic wide knockout screening identifies mitochondrial ribosomal proteins involving in oxygen‐glucose deprivation/reperfusion resistance
Source: J Cell Mol Med. 2020 Jul 2;24(16):9313–22. doi: 10.1111/jcmm.15580 (PMC7417733; doi:10.1111/jcmm.15580)
Supplement: Supplementary file 2 — Supplementary Material [file JCMM-24-9313-s002.docx]

Table S2 Protein-protein interaction network and MCODE components identified in the gene lists.

|  | MCODE | GO | Description | Log10(P) |
| --- | --- | --- | --- | --- |
|  | MCODE_1 | GO:0051204 | protein insertion into mitochondrial membrane | -9.5 |
|  | MCODE_1 | hsa04215 | Apoptosis - multiple species | -9.5 |
|  | MCODE_1 | GO:0051205 | protein insertion into membrane | -8.6 |
|  | MCODE_2 | R-HSA-5637815 | Signaling by Ligand-Responsive EGFR Variants in Cancer | -8.9 |
|  | MCODE_2 | R-HSA-1236382 | Constitutive Signaling by Ligand-Responsive EGFR Cancer Variants | -8.9 |
|  | MCODE_2 | R-HSA-8856828 | Clathrin-mediated endocytosis | -8.8 |
|  | MCODE_3 | CORUM:320 | 55S ribosome, mitochondrial | -20.1 |
|  | MCODE_3 | R-HSA-5389840 | Mitochondrial translation elongation | -19.7 |
|  | MCODE_3 | R-HSA-5419276 | Mitochondrial translation termination | -19.7 |
|  | MCODE_4 | R-HSA-5617833 | Cilium Assembly | -6.8 |
|  | MCODE_4 | R-HSA-1852241 | Organelle biogenesis and maintenance | -6.1 |
|  | MCODE_4 | GO:0097711 | ciliary basal body-plasma membrane docking | -5.7 |
|  | MCODE_5 | GO:0006790 | sulfur compound metabolic process | -9.1 |
|  | MCODE_5 | GO:0051923 | sulfation | -8.6 |
|  | MCODE_6 | hsa04080 | Neuroactive ligand-receptor interaction | -7.8 |
|  | MCODE_6 | R-HSA-500792 | GPCR ligand binding | -6.9 |
|  | MCODE_6 | R-HSA-418555 | G alpha (s) signalling events | -6.6 |
|  | MCODE_7 | R-HSA-381340 | Transcriptional regulation of white adipocyte differentiation | -6.8 |
|  | MCODE_7 | R-HSA-1989781 | PPARA activates gene expression | -6.4 |
|  | MCODE_7 | R-HSA-400206 | Regulation of lipid metabolism by Peroxisome proliferator-activated receptor alpha (PPARalpha) | -6.3 |
